# SETDB1 promotes gastric cancer progression via UPR and mTOR pathway

**DOI:** 10.1186/s12935-026-04296-1

**Published:** 2026-04-11

**Authors:** Jing Qiao, Shijie Lin, Yeju Li, Xiaoyang Yue, Yanyan Liu, Huajian Tian, Jianshuang Li, Junyang Tan

**Affiliations:** 1 The Affiliated Qingyuan Hospital (Qingyuan People’s Hospital), Guangzhou Medical University, Qingyuan, 511518 Guangdong China; 2 State Key Laboratory of Bioactive Molecules and Druggability Assessment, Guangdong Basic Research Center of Excellence for Natural Bioactive Molecules and Discovery of Innovative Drugs, College of Life Science and Technology, Jinan University, Guangzhou, 510632 Guangdong China; 3 Department of Anesthesiology and Clinical Research Institute, The First Affiliated Hospital of Jinan University, Guangzhou, 510630 Guangdong China; 4 Department of Orthopaedics, Guangzhou Red Cross Hospital, Faculty of Medical Science, Jinan University, Guangzhou, 510220 Guangdong China

**Keywords:** Gastric cancer, SETDB1, Histone H3K9 methyltransferase, Unfolded protein response, MTOR

## Abstract

SET Domain Bifurcated histone lysine methyltransferase 1 (SETDB1), a histone H3K9 methyltransferase, is frequently overexpressed in multiple malignancies, including gastric cancer (GC). However, its molecular functions and regulatory mechanisms in GC progression remain poorly defined. Here, we identified the role of SETDB1 in gastric cancer using molecular and cell biology methods, combining RNA-seq and RNC-mRNA seq. Here, we found SETDB1 was markedly upregulated in gastric cancer tissues compared with adjacent non‑tumor tissues. Functional analyses revealed that SETDB1 knockdown suppressed GC cell proliferation and migration, whereas overexpression had the opposite effect. Notably, SETDB1 promoted cell proliferation independently of its methyltransferase activity, while its enhancement of migration required enzymatic function. Mechanistically, SETDB1 activated the unfolded protein response (UPR) to stimulate c-MYC transcription and cell-cycle progression, and engaged the mTOR–4EBP1 axis to promote HIF1α translation. These findings reveal a dual regulatory role for SETDB1 in GC and identify it as a promising therapeutic target for managing gastric cancer.

**Supplementary Information:**

The online version contains supplementary material available at 10.1186/s12935-026-04296-1.

## Introduction

Globally, more than 650, 000 people succumb to gastric cancer annually, placing it fifth in terms of both mortality and morbidity [1]. Most gastric cancer (GC) patients are diagnosed at advanced stage and their treatment typically involves surgical resection and chemotherapy, with poor prognosis [2, 3]. Therefore, a better understanding of the molecular mechanisms driving GC progression is crucial for identifying novel therapeutic targets. GC arises from a complex interplay of environmental, dietary, and genetic factors *Helicobacter pylori (H. pylori)* infection, high salt diet, smoked or poorly preserved food, tobacco smoking, alcohol consumption, and hereditary predisposition are established risk factors. Beyond genetic mutations, epigenetic alterations—including DNA methylation, histone modifications, and dysregulated miRNA expression—also contribute to GC initiation and progression, though the underlying mechanisms remain poorly defined.

Histone modifications are covalent post-translational modification to histone tails which including acetylation, methylation, phosphorylation, sumoyation and ubiquitylation [4]. These modifications influence transcriptional activity, chromatin configuration, replication, and oncogenic transformation [5]. Histone methylation is a process that methyl group (CH3) are added to the lysine or arginine residues by the histone methyltransferase (HMT), and it can be reversed by the histone demethylase [6]. Dysregulated histone methyltransferases and demethylases are increasingly recognized as contributors to GC pathogenesis. Among HMTs, Enhancer of Zeste Homolog 2 (EZH2) catalyzes H3K27 trimethylation and promotes GC progression by silencing tumor‑suppressor genes. Elevated EZH2 expression correlates with aggressive clinicopathological features and poor survival, and several EZH2 polymorphisms (SNP; rs12670401, rs6464926, rs2072407, rs734005, and rs734004) are associated with gastric cancer susceptibility. BMI1, a core component of the Polycomb repressive complex 1 that recognizes H3K27me3, is overexpressed in GC and promotes invasion and metastasis [7, 8]. Among histone demethylases, JMJD2B and RBP2 also involved in gastric tumorigenesis. JMJD2B, specifically target trimethylated H3K9, is required for Helicobacter pylori-induced gastric carcinogenesis via regulating COX-2 expression [9]. Moreover, JMJD2B promotes EMT and gastric cancer invasion and metastasis by cooperating with β-Catenin [10]. Retinoblastoma binding protein 2 (RBP2), a newly demethylase for H3K4 trimethylation and demethylation, promotes gastric tumorigenesis by transactivating VEGF expression and elevating angiogenesis [11]. RBP2 also promotes GC malignant progression through TGF-β1-(p-Smad3)-RBP2- E-cadherin-Smad3 feedback circuit and its epigenetic inhibition of cyclin-dependent kinase inhibitors [12, 13].

SET Domain Bifurcated histone lysine methyltransferase 1 (SETDB1) is another H3K9‑specific histone methyltransferase belonging to the SET‑domain family. It catalyzes the di‑ and trimethylation of H3K9 [14, 15]. and participates in diverse biological processes, including cell proliferation, epigenetic silencing, promyelocytic leukemia nuclear body (PML‑NB) assembly, retroelement repression, and control of cell‑cycle arrest [16–20]. Accumulating evidence indicates thatSETDB1 is overexpressed in multiple cancers, including gastric cancer, liver cancer, colorectal cancer and breast cancer [16, 20–22], suggesting that dysregulated SETDB1 contributes to oncogenesis. However, its precise molecular function and regulatory mechanisms in GC remain unclear, warranting further investigation.

In this study, we demonstrate that SETDB1 promotes gastric cancer cell proliferation and migration. Specifically, SETDB1‑driven proliferation occurs independently of its methyltransferase activity, whereas migration requires enzymatic function. Mechanistically, SETDB1 knockdown suppressed c‑MYC transcription by impairing UPR activation, thereby blocking cell‑cycle progression. Moreover, SETDB1 was found to enhance HIF1α translation through the mTOR–4EBP1 signaling axis. Together, these findings identify SETDB1 as a key epigenetic regulator linking histone modification, UPR signaling, and translational control, and establish it as a potential therapeutic target in gastric cancer.

## Materials and methods

### Cell culture

 293 T (SCSP-502), AGS (TCHu232) and SGC-7901 (TCHu46) cells were obtained from the Type Culture Collection Preservation Committee, Chinese Academy of Sciences (Shanghai, China). All the cells were cultured in Dulbecco’s Modified Eagle Medium (DMEM) containing 10% fetal bovine serum and 1% penicillin and streptomycin at 37 °C under 5% CO_2_ conditions, 95% humidity.

### Plasmids

For the design of shRNA targeting SETDB1, the BLOCK-iT™RNAi Designer (http://rnaidesigner.thermofisher.com/) was employed to identify the optimal shRNA sequences. Two target sequences are as follows:

shSETDB1-1: 5′-CCCGAGGCTTTGCTCTTAAA-3′;

shSETDB1-2: 5′-AGTTAGAGACATGGGTAATAC-3′;

All the target sequences for shRNA were cloned into pLKO.1 vector according to according to our previous protocol [23].

For overexpression plasmid, the pLenti CMV GFP Puro vector was digested with XbaI and NotI to linearize the vector and then ligated SETDB1 coding sequence and its double site methyltransferase-dead mutation (H1224K and C1226A) with T4 ligase.

### Lentivirus package and transfection

In brief, specific plasmids were transfected into 293 T cells with psPAX2 and pMD2.G using polyethyleneimine (PEI) transfection reagent. Viral supernatants were respectively collected at 48 and 72 h post-transfection and utilized to infect target cells in the presence of 10 µg/mL polybrene. Following 24 h, the medium was supplemented with 1–2 µg/mL puromycin to select for the selection of stably infected cells.

### Western blotting

The cells were lysed using ice-cold RIPA buffer with PMSF and phosphatase inhibitor. Following the determination of protein concentrations by the bicinchoninic acid assay (BCA), the cell lysates were combined with 5 × SDS-loading buffer and subjected to boiling at 95 °C for 10 min. Each protein sample was loaded and fractionated by SDS-PAGE gels and subsequently transferred onto PVDF membranes. The membranes were then blocked for 1 h with 5% skim milk, followed by incubation with specific primary and HRP-conjugated secondary antibodies. Immunoreactive bands were visualized using chemiluminescence detection reagents, and the intensities of the bands were quantified by Image J.

### Quantitative real-time PCR

Total RNA was isolated using the RNAiso Plus reagent (#9109, TaKaRa) by the manufacturer’s protocol. Subsequently, one microgram of the purified total RNA was reverse transcribed into cDNA using the ABScript II cDNA First Strand Synthesis Kit (RK20400, ABclonal). The relative expression of diverse genes was quantified by real-time PCR employing the SYBR Green Master Mix (RK21203, Abclonal) on a CFX96 real-time detection system (BioRad, Hercules, CA). Actin beta (ACTB) was employed as an internal control for normalization, and the results were determined using the 2^−ΔΔCt^ method. Primer sequences for target genes are shown in Supplementary Table 2.

### Immunofluorescence

The cells were fixed with 4% paraformaldehyde for 15 min after reaching 60% confluence on coverslips. The fixed coverslips were rinsed three times with PBS and permeabilized with 0.2% Triton X-100 for 10 min. Afterward, the cells were blocked with 5% bovine serum albumin. The blocked cells were incubated with primary antibodies overnight at 4 °C followed by the secondary antibodies for 1 h protected from light at room temperature (Supplementary Table 1). Cell nuclei were visualized by counterstaining with 4 ´,6-diamidino-2phenylindole (DAPI). After rinsing with PBS, the cells were mounted with an anti-fluorescent quenching sealing agent (P0128S, Beyotime) and imaged using a Leica TCS SP8 confocal microscope.

### EDU staining assay

For the detection of cell proliferation, each cell group was stained in accordance with the instructions provided by the BeyoClick™ EdU Cell Proliferation Kit with Alexa Fluor 594 (C0078S; Beyotime). Specifically, 5 × 10^4^ cells were seeded in 12-well plates. Following a 24-hour incubation in complete medium, the EDU staining solution was applied and incubated for 2 h. Subsequently, the cells were fixed with 4% paraformaldehyde (PFA) for 15 min, permeabilized with 0.2% Triton X-100 for 10 min, incubated with Click Additive Solution for 30 min, and then stained with Hoechst 33,342 for 10 min for nuclei visualization. Washed with detergent after each operation procedure. Images were captured with Nikon Ti2-U Fluorescence microscope.

### Wound-healing, migration, and invasion assays

Cell migration and invasion were evaluated using previously described methods. For scratch wound-healing assays, cell groups were seeded into 6-well plates and cultured until reaching 90% confluence. To inhibit cell proliferation during migration, serum starvation was initiated 12–16 h prior to creating the wound scratch on the cell monolayer, with maintenance in Low-serum medium (1% FBS) throughout the experiment. After gentle washed with PBS to remove floating cells, monitoring was conducted using inverted microscopy at 24-hour intervals.

For transwell migration and invasion assays, cells were subjected to 1% serum starvation for 16 h prior to cell counting. AGS (5 × 10^4^) or SGC-7901 (8 × 10^4^) cells were plated into 24-well Boyden chambers (for migration) or Matrigel-coated invasion chamber (for invasion) with 8.0 μm polycarbonate membrane (#3422, Corning) after trypsinized and suspended in Low-serum medium (1% FBS), while 600 µL medium containing 20% FBS was filled in the bottom of wells. The plates were then cultured for 24–48 h, allowing cells that migrated through the membrane to be fixed with 4% paraformaldehyde for 15 min and stained with a 0.1% crystal violet solution for 10 min at room temperature. The non-migrated cells on the top layer of the membrane were removed using a cotton swab. The membranes were air-dried after extensive wash with water, and then the migrated cells were counted under a microscope. At least five random fields in each group were imaged and analyzed in the aforementioned experiments.

### RNA-sequencing and RNC-sequencing analysis

For RNA-sequencing, total RNA was extracted using Trizol reagent. The RNA was then sequenced by the Novegene RNA-sequencing service (*n* = 3). GO analysis of the Differentially Expressed Genes significant changes was performed using Metascape website. For gene set enrichment analysis, we applied GSEA v4.1.0 to various functional characteristic gene signatures as described previously. GSEA was performed using the “Hallmark” or “KEGG” gene sets to identify enriched signatures. Gene Sets with an FDR < 0.25 and a nominal p-value of < 0.05 were considered significant.

For RNC-sequencing, the samples were sequenced by the chi-biotech RNC-sequencing service (*n* = 3). The analysis method has been stated in the previous work [24].

### Colony formation and CCK-8 assays

For colony formation, about 1 × 10^3^ pLKO.1 and shC11orf54 PLC/PRF/5 cells were seeded in a six-well plate and treated with the indicated concentration of cisplatin. The cells were cultured for 10 days, and 4% paraformaldehyde was used to fix the cells, followed by staining with 0.5% crystal violet for 1 h. The number of colonies (> 50cells/colony) was counted using a stereomicroscope and analyzed by image J software. All the samples were done in triplicate.

CCK-8 (Solarbio) was used to assess the cell proliferation following the manufacturer’s instructions. Briefly, cells were seeded in 96-well plates at the density of 1 × 10^3^ cells/well, then cultured in an incubator for 24 h before being evaluated at day-1, −2, −3, −4, −5, respectively. CCK-8 solution was then dripped into each well, and the plate was transferred to the incubator for two hours. Finally, an OD value at 450 nm was detected by MD SpectraMax 190.

### Statistics and reproducibility

All statistical analyses were performed using Excel (Microsoft) or Prism (GraphPad 8.0) software. The statistical results are presented as the mean ± standard deviation (S.D.), and *p*-values were calculated using unpaired two-tailed Student’s *t*-test for pairwise comparisons. *P* < 0.05 was considered as statistical significance (* indicates *P* < 0.05; ** indicates *P* < 0.01; *** indicates *P* < 0.001). All experiments were performed independently at least three times unless stated otherwise in the figure legend.

## Results

### SETDB1 is upregulated in GC and promotes GC cells proliferation in a methyltransferase activity-independent manner

To investigate the role of histone methylation in the development of gastric cancer, we evaluated the alteration and expression of five H3K9 methyltransferases (SETDB1, SUV39H1, SUV39H2, EHMT1, EHMT2) in gastric cancer samples. The proportion and distribution of altered samples indicated that SETDB1 and EHMT2 were the two most frequently altered genes among the H3K9 methyltransferases, appearing in 30 out of 393 (8%) stomach adenocarcinoma cases according to cBioPortal (www.cbioportal.org) (Figure S1A). Moreover, our analyses of the TCGA database revealed that all five genes were significantly upregulated in tumor tissues compared with the adjacent normal tissues (Figure S1B). Furthermore, Kaplan–Meier and log-rank analyses further indicated that high mRNA levels of SETDB1, SUV39H1, and EHMT2, together with decreased expression of SUV39H2 and EHMT1, were significantly correlated with poorer overall survival (OS) in patients with gastric cancer (Figure S1C-G).

**Fig. 1:**
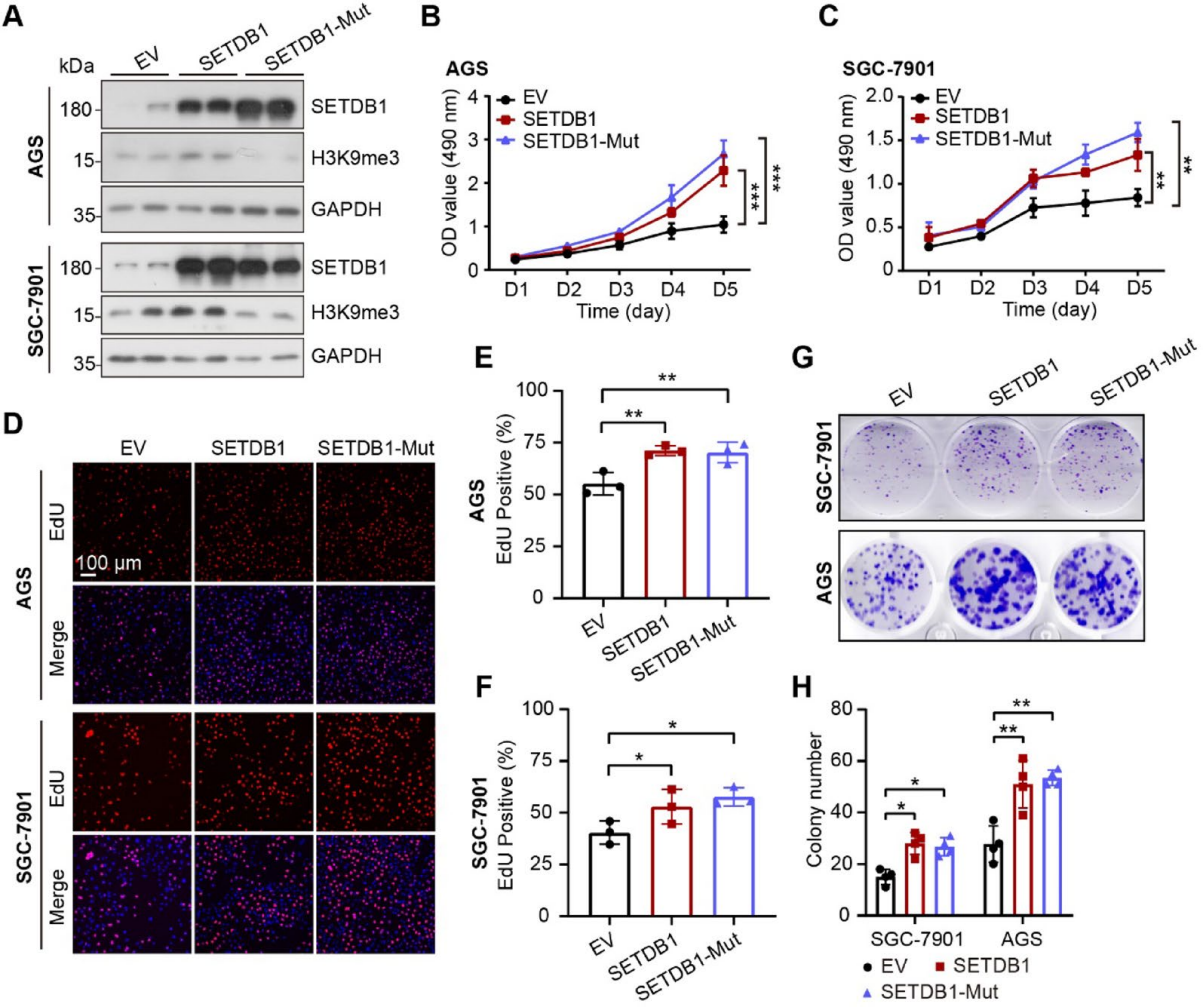
SETDB1 promotes the proliferation of GC cells. **A.** Representative western blot results of SETDB1 and H3K9me3 in AGS and SGC-7901 cell lines. **B-C.** CCK8 assay shows the cell survival of SETDB1 and SETDB1-Mut in AGS and SGC-7901 cell lines (data are presented as mean values ± SD, **p < 0.01, ***p < 0.001). **D-F.** Representative images of EdU staining (**D**) and quantification results (**E-F**) in AGS and SGC-7901 cells. The images display EdU staining (red color) merged with DAPI staining (blue color) (Scale bar = 100 μm; data are presented as mean values ± SD, *p < 0.05, **p < 0.01). **G-H.** Colony formation analysis of SETDB1 and SETDB1-Mut in AGS and SGC-7901 cell lines (data are presented as mean values ± SD, *p < 0.05, **p < 0.01). EV: empty vector, the control cells; SETDB1: SETDB1 stably overexpressed cells; SETDB1-Mut: methyltransferase dead mutant SETDB1 (H1224K/C1226A) stably overexpressed cells

To explore the functional role of SETDB1 in the progression of GC, we conducted loss-of-function assays using two gastric adenocarcinoma cell lines. SETDB1 knockdown significantly inhibited GC cell proliferation and clonogenic capacity (Figure S2). In contrast, the overexpression of SETDB1 promoted the proliferation of GC cells (Fig. 1A-C). To determine whether SETDB1’s promotion of GC cell proliferation was dependent on its methyltransferase activity, we generated a methyltransferase activity-deficient mutant of SETDB1 (SETDB1-H1224K/C1226A, referred to as SETDB1-Mut). Our findings revealed that, in comparison to the wild-type SETDB1, SETDB1-Mut did not increase the expression of H3K9me3, confirming the absence of methyltransferase activity in SETDB1-Mut (Fig. 1A). Notably, both wild-type and mutant SETDB1 promoted GC cell proliferation and colony formation (Fig. 1D-H). Collectively, these data suggest that SETDB1 is upregulated in gastric cancer, correlates with poor prognosis, and promotes GC cell proliferation independently of its methyltransferase activity.

### SETDB1 regulates the gastric cancer cell cycle by modulating c-MYC transcription

**Fig. 2:**
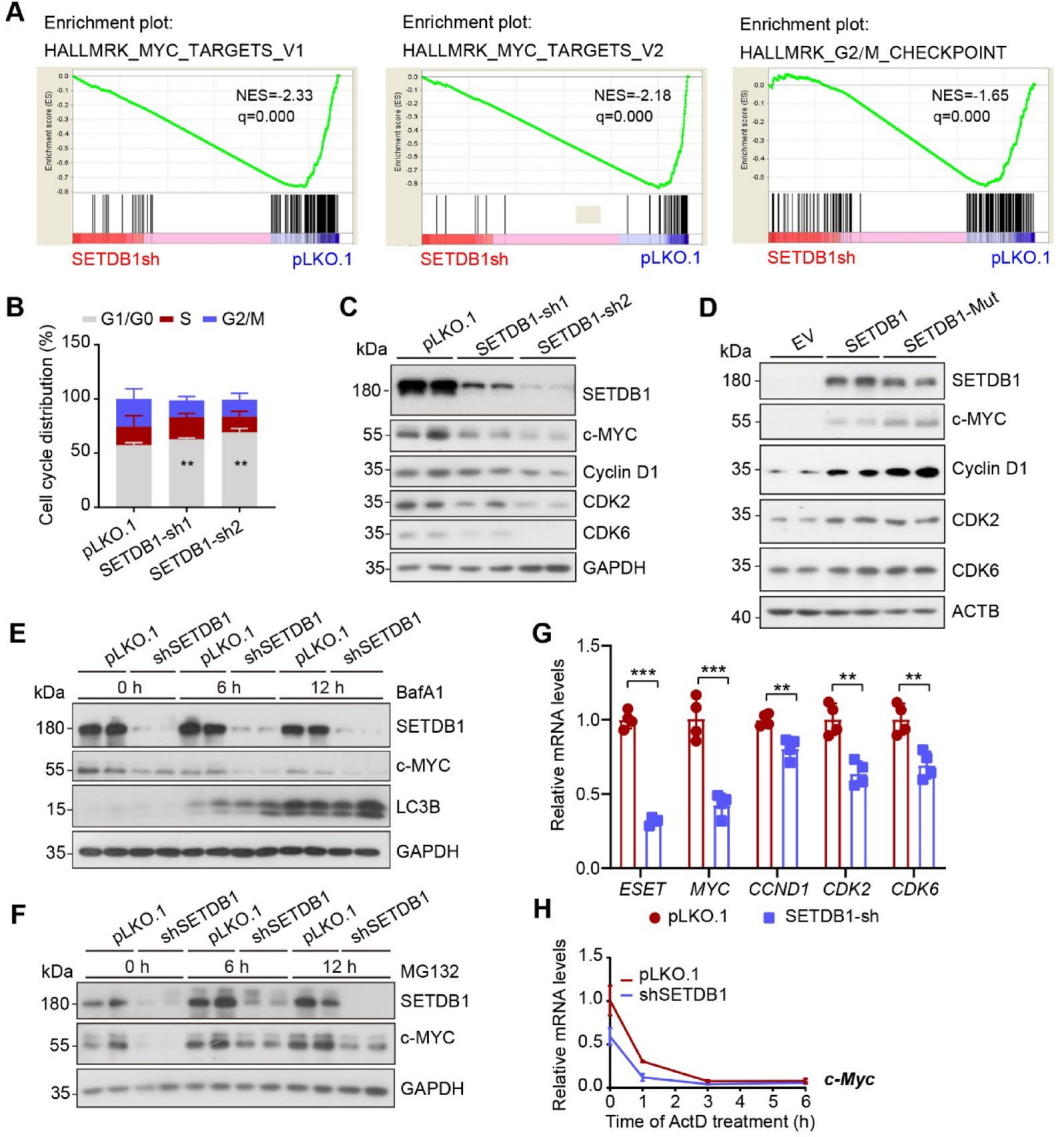
Knockdown of SETDB1 represses the cell cycle of gastric cancer cells by suppressing the transcriptional levels of c-MYC. **A.** Gene set enrichment analysis (GSEA) shows MYC targets and G2/M checkpoint signaling pathways were enriched in the SETDB1 knockdown group. **b** Flow cytometry experiments analyze the cell cycle in control and SETDB1 knockdown cells. (data are presented as mean values ± SD, **p < 0.01). **C-D.** Representative Western blots of the indicated proteins in SETDB1 knockdown (**C**) and overexpressed (**D**) cells. **E-F.** Representative western blots of the indicated proteins in control and SETDB1 knockdown cells upon 100 nM BafA1 (**E**) or 10 µM MG132 (**F**) treatment for 6 and 12 h. **G.** qPCR analysis of the mRNA expression of MYC and its downstream cell cycle related genes in SETDB1 knockdown and control cells (data are presented as mean values ± SD, **p < 0.01, ***p < 0.001). **H.** c-MYC mRNA stability in control and SETDB1 knockdown cells assessed by qPCR following 5 µg/ml Actinomycin D (ActD) treatment

To elucidate the underlying mechanisms by which SETDB1 promotes GC proliferation, we performed RNA sequencing on SETDB1-knockdown cells and control cells. Gene Set Enrichment Analysis (GSEA) revealed that MYC target genes and G2/M checkpoint-related pathways were significantly downregulated after SETDB1 depletion (Fig. 2A). Flow cytometry was used to assess cell cycle progression and validated the impact of SETDB1 depletion on the cell cycle. The results indicated a significant increase in the proportion of cells in the G1/G0 phase after SETDB1 knockdown (Fig. 2B). Subsequently, we examined the expression levels of c-MYC and its downstream cell cycle-related proteins. Western blot analysis showed that silencing SETDB1 inhibited the protein expression of c-MYC and associated cell cycle proteins (Fig. 2C). Conversely, overexpression of SETDB1 or its methyltransferase mutant led to an increase in the expression of c-MYC and its targeted cell cycle proteins (Fig. 2D).

To investigate the underlying causes of the reduction in c-MYC level, we initially treated the cells with autophagy and proteases inhibitors separately to assess c-MYC degradation. However, no evidence of c-MYC protein degradation was detected under either BafA1 or MG132 treatment conditions (Fig. 2E-F). Instead, we observed a significant downregulation of the c-MYC gene and its downstream target genes at the mRNA level (Fig. 2G). Further validation using Actinomycin D (ActD) demonstrated that the inhibition of c-MYC protein resulting from SETDB1 knockdown was due to transcriptional regulation (Fig. 2H). Collectively, these findings confirm that SETDB1 regulates the cell cycle by modulating c-MYC transcription.

### SETDB1 modulates the transcription of c-MYC through UPR pathway

**Fig. 3:**
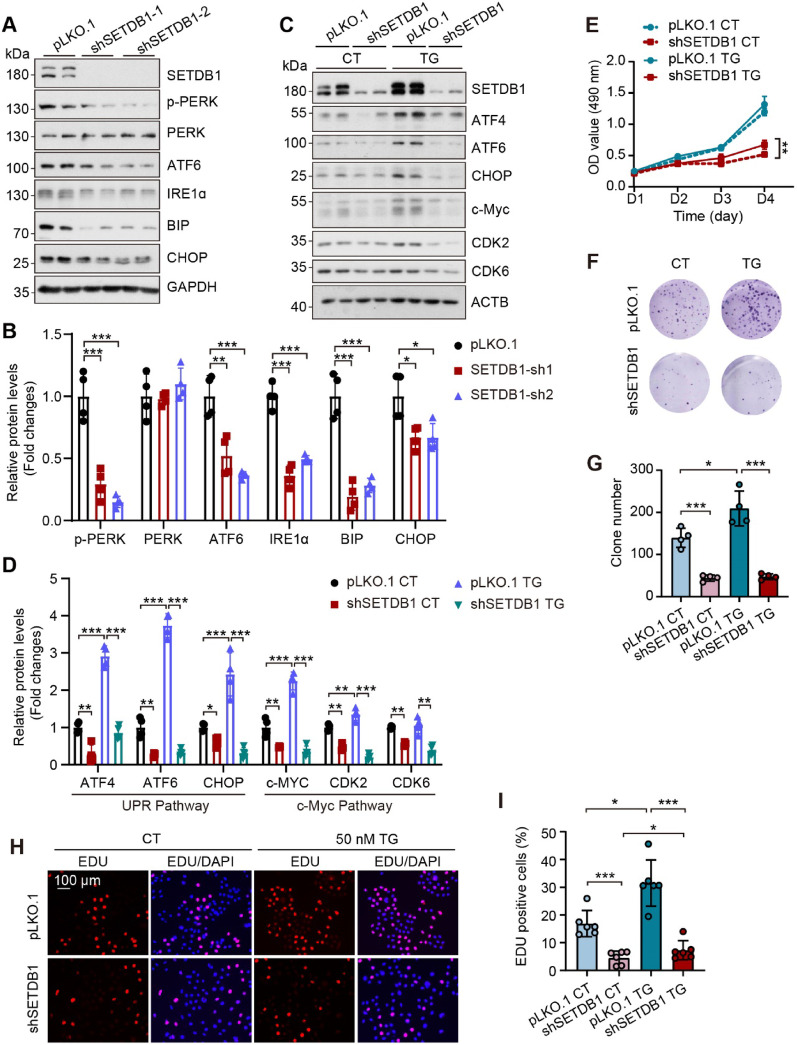
Knockdown of SETDB1 represses gastric cancer cells proliferation via the suppression of UPR. **A-B.** Representative western blots (**A**) and quantitative results (**B**) of the indicated proteins in the control and SETDB1 knockdown AGS cells. (data are presented as mean values ± SD, *p < 0.05, **p < 0.01, ***p < 0.001) **C-D.** Representative western blots (**C**) and quantitative results (**D**) of the indicated proteins in the control and SETDB1 knockdown AGS cells upon 100 nM Thapsigargin (TG) treatment for 12 h. **E.** CCK8 assay analyzed cell survival of the control and SETDB1 knockdown cells upon 100 nM Thapsigargin (TG) treatment for 12 h (data are presented as mean values ± SD, **p < 0.01, ***p < 0.001). **F-G.** Colony formation analysis of the control and SETDB1knockdown AGS cells upon 100 nM Thapsigargin (TG) treatment. (data are presented as mean values ± SD, *p < 0.05, **p < 0.01). **H-I.** Representative EdU staining and quantitative results in the control ad SETDB1 knockdown AGS cells upon 100 nM Thapsigargin for 12 h. The images display EdU staining (red color) merged with DAPI staining (blue color) (Scale bar = 100 μm; data are presented as mean values ± SD, *p < 0.05, ***p < 0.001)

Recent studies have indicated that the unfolded protein response (UPR) pathway is involved in the regulation of c-MYC transcription and expression [25]. Given the critical role of UPR in the progression of gastric cancer [26], we investigated the protein expression levels within the UPR pathway following the knockdown SETDB1. Western blot analysis demonstrated that the depletion of SETDB1 resulted in the inhibition of sensors and downstream proteins expression within the UPR pathway (Fig. 3A-B). To further explore this finding, we employed Thapsigargin (TG), a well-established UPR inducer, to activate the unfolded protein response in SETDB1 knockdown cells. Our observations revealed that TG treatment significantly upregulated the expression of UPR pathway proteins and c-MYC; however, this upregulation was markedly suppressed in the absence of SETDB1 (Fig. 3C-D). Moreover, TG treatment enhanced proliferation and colony-forming capacity in control cells, where the effects were not observed in SETDB1 knockdown cells (Fig. 3E-I). The pLKO.1 group exhibited muted survival response to TG, likely reflecting inherent resistance to UPR activation in this cellular context. Nevertheless, shSETDB1 consistently reduced survival under TG stress, underscoring its pro-survival role.

**Fig. 4:**
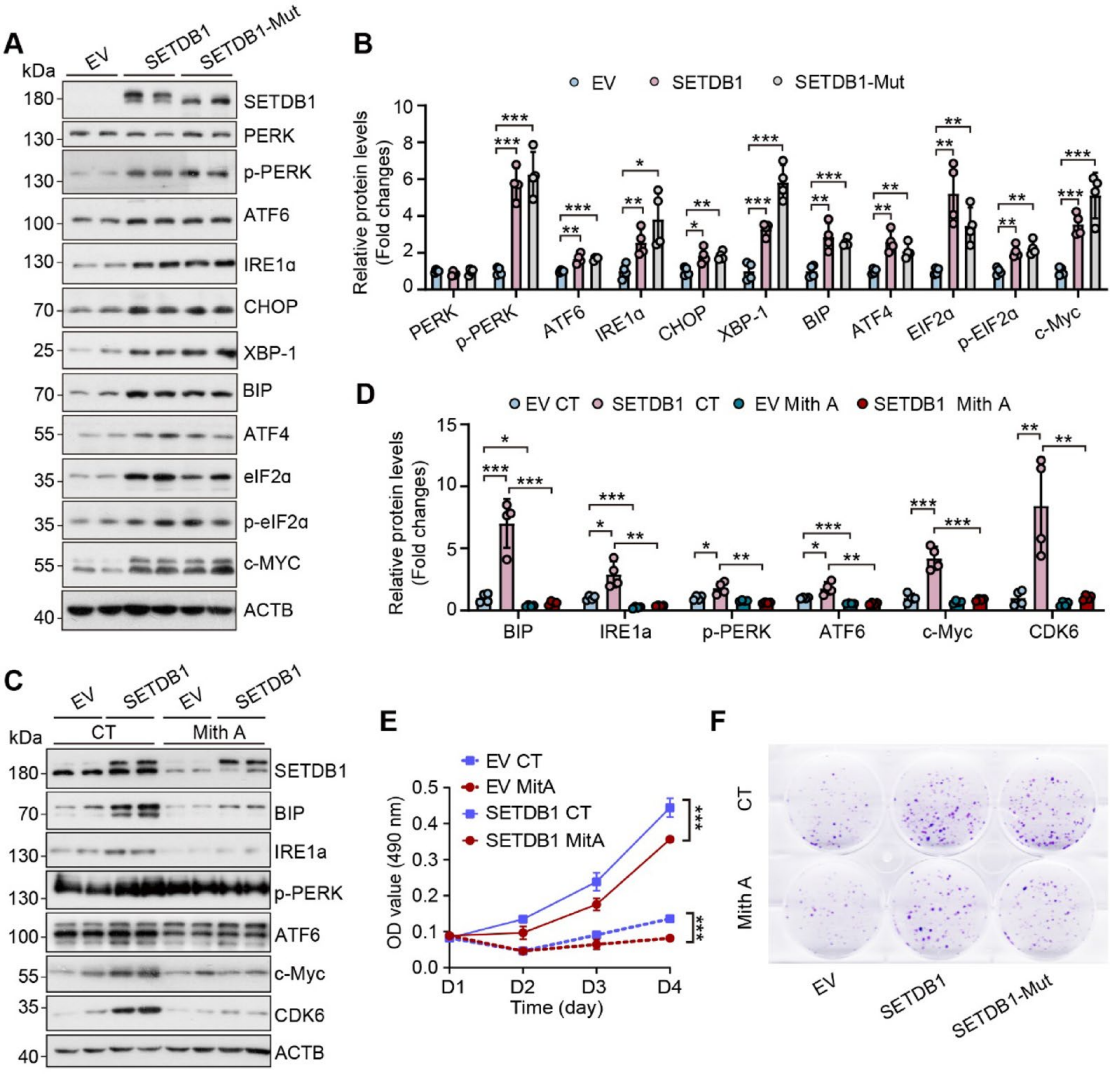
Overexpression of SETDB1 activated UPR and c-Myc expression independent of its methyltransferase activity. **A-B.** Representative western blots (**A**) and quantitative results (**B**) of the indicated proteins in SETDB1 and SETDB1-Mut cells (data are presented as mean values ± SD, *p < 0.05, **p < 0.01, ***p < 0.001).**C-D.** Representative western blots (**C**) and quantitative results (**D**) of the indicated proteins in control and SETDB1/SETDB1-Mut overexpression cells upon 100nM Mithramycin A (Mith A) treatment for 24 h(data are presented as mean values ± SD, *p < 0.05, **p < 0.01, ***p < 0.001). **E.** CCK8 assay analyzed the cell survival of control and SETDB1 overexpression cells upon 100nM Mithramycin A (Mith A) pretreatment for 24 h (data are presented as mean values ± SD, **p < 0.01, ***p < 0.001). **F.** Colony formation analysis of control and SETDB1 overexpression cells upon Mithramycin A (Mith A) treatment

Subsequently, we examined the effects of overexpressing SETDB1 and its methyltransferase activity-null mutant on UPR-related proteins. Our findings indicated that increased levels of SETDB1 augmented the expression of UPR pathway related proteins, and this modulation occurred independently of its enzymatic activity (Fig. 4A-B). These observations consistent with our previous findings regarding the proliferation-promoting effects of SETDB1 were independent of its enzymatic function. Furthermore, we employed Mithramycin A (Mith A), a previously characterized inhibitor of SETDB1 [27, 28] to explore the relationship between SETDB1 and UPR pathway proteins as well as c-MYC. The results revealed that Mith A inhibited the expression of SETDB1, leading to a subsequent downregulation of UPR pathway proteins, c-MYC, and its associated cell cycle proteins (Fig. 4C-D). Additionally, Mith A treatment suppressed the proliferation and clonogenic capacity of gastric cancer cells (Fig. 4E-F). These results suggest that SETDB1 regulates c-MYC expression via the UPR pathway, thereby promoting the proliferation of gastric cancer cells.

### SETDB1 facilitates GC cell migration in a methyltransferase activity-dependent manner

**Fig. 5:**
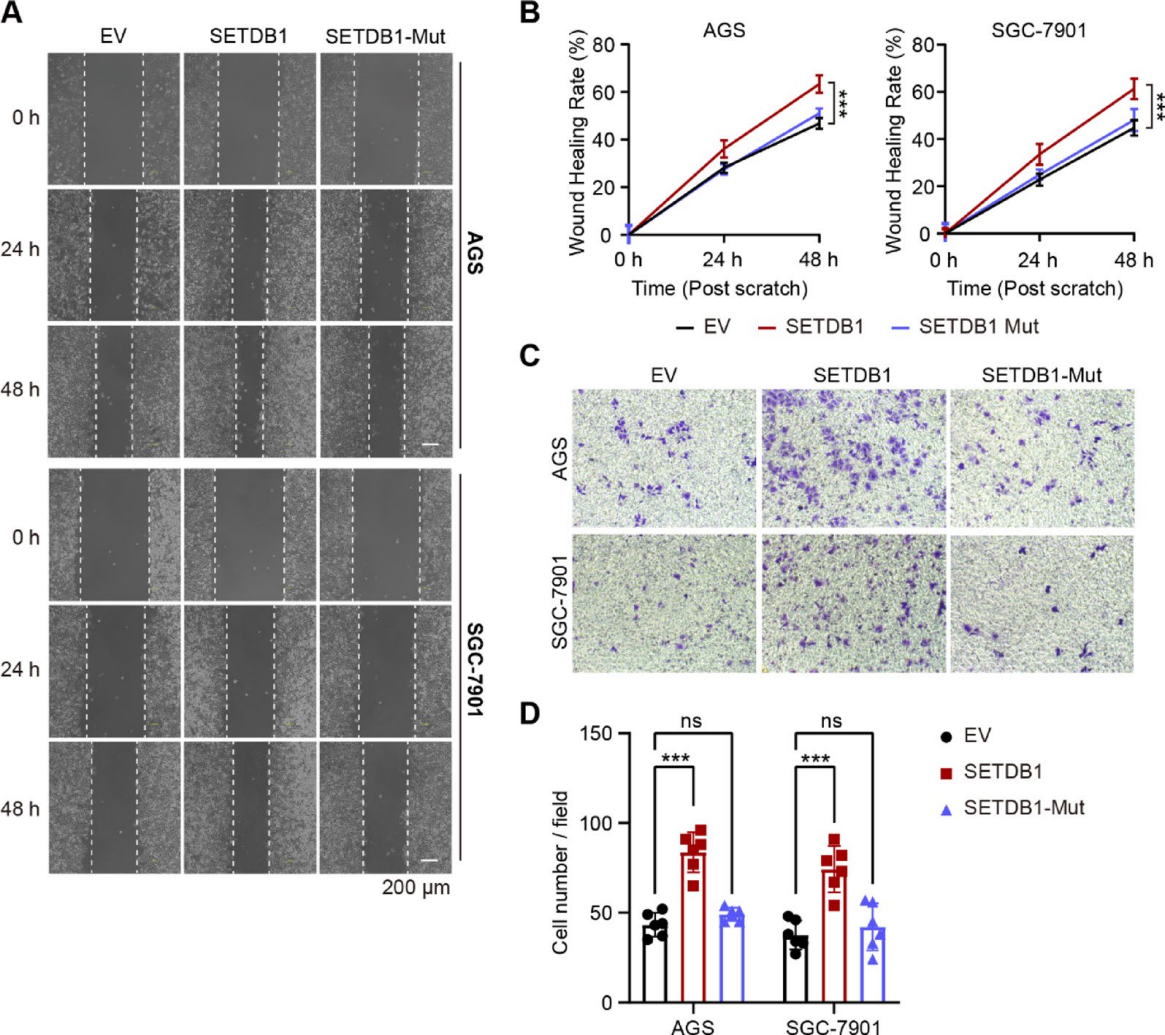
SETDB1 promotes gastric cancer cell migration dependent on its methyltransferase activity. **A-B.** Representative images (**A**) and wound healing rate (**B**) of the wound healing assay in SETDB1 and SETDB1-Mut AGS and SGC-7901 cells (Scale bar = 200 μm; data are presented as mean values ± SD, **p < 0.01, ***p < 0.001).**C-D.** Trans-well assay (**C**) and statistical results (**D**) in SETDB1 and SETDB1-Mut AGS and SGC-7901 cells (data are presented as mean values ± SD, ***p < 0.001)

Next, we investigated the potential involvement of SETDB1 in the migration of GC cells. Both wound healing and trans-well assays demonstrated that the depletion of SETDB1 led to a reduction in the migratory capacity of GC cells (Figure S3). Conversely, the overexpression of SETDB1 resulted in an enhanced migration rate of these cells (Fig. 5). Importantly, our findings revealed that the methyltransferase activity of SETDB1 is crucial for the migration of GC cells, which is contrasts with the previous studies that focused on the solitary mutation of H1224K [29]. When the double site methyltransferase-dead mutation of SETDB1 (H1224K and C1226A) was overexpressed, the migratory ability of gastric cancer cells remained unchanged. These results suggest that SETDB1 promotes the migration of gastric cancer cells through its methyltransferase activity.

### SETDB1 regulates the expression of HIF1α at the translational level

**Fig. 6:**
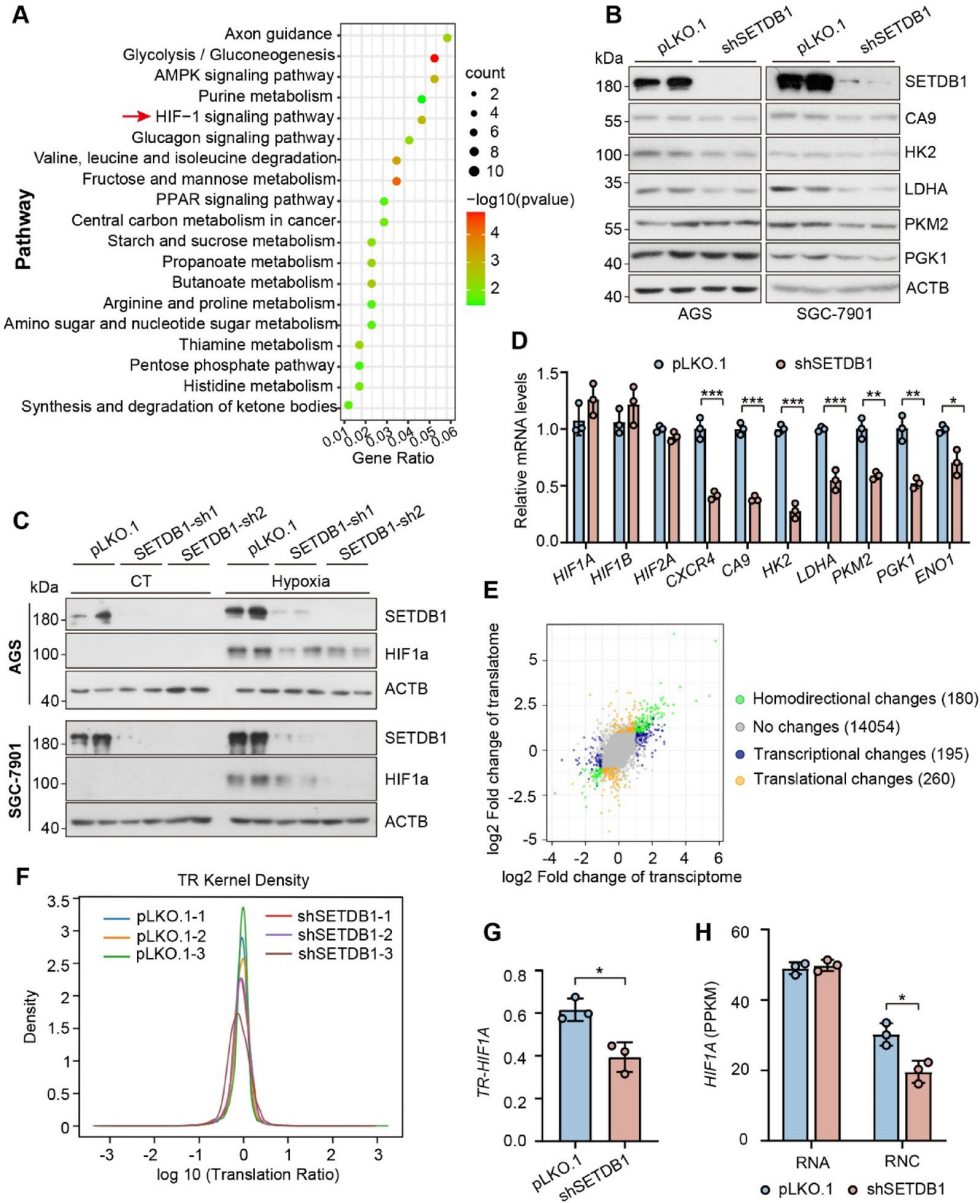
SETDB1 regulates the expression of HIF1α at the translational level. **(A)** The top 20 functionally enriched KEGG pathways found in the analysis of DEGs in SETDB1 knockdown versus control sample. **(B)** Representative western blots of the indicated proteins of control and SETDB1 knockdown in AGS and SGC-7901 cell lines. **(C)** Representative western blots of the indicated proteins of control and SETDB1 knockdown in AGS and SGC-7901 cell lines upon 12 h hypoxia treatment. **(D)** qPCR analysis of the mRNA expression of *HIF1* and its downstream genes in SETDB1 knockdown and control cells (data are presented as mean values ± SD, *p < 0.05, **p < 0.01, ***p < 0.001). **(E)** Nine quadrant map of common differential genes between transcriptome and translatome. **(F)** The TR Kernel density estimation in control and SETDB1 knockdown cells. **G-H.** Translation ratio (TR) and ribosome nascent-chain complex (RNC) of *HIF1Α* (data are presented as mean values ± SD, *p < 0.05)

To explore how SETDB1 regulate the proliferation and migration of gastric cancer cell, we performed functional analyses of DEGs (Differentially Expressed Genes) using the KEGG database and found that HIF1α signaling pathway was one of the most significantly downregulated pathways (Fig. 6A). As an oxygen sensitive transcription factor, studies have proved that overexpression of HIF1α is frequently observed in gastric cancer and is linked to poor prognosis, including reduced overall survival and increased metastasis [30–33]. In this study we confirmed that loss of SETDB1 suppressed the expression of HIF1α targets, such as CA9, HK2, LDHA, PKM2 and PGK1 in AGS and SGC-7901 cells (Fig. 6B). However, SETDB1-knockdown-mediated suppression of HIF1α was still existed under hypoxia condition (Fig. 6C). Furthermore, suppressed HIF1α was also observed even under two distinct prolyl hydroxylase inhibitors (DMOG and CoCl_2_) treatment in the SETDB1 knockdown GC cells (Figure S4A-B). These results suggested that SETDB1 regulated HIF1α may not at the protein level. Then we examined the mRNA level and found that the knockdown of SETDB1 repressed the mRNA levels of HIF1α target genes, but with no changes in the mRNA levels of *HIF1Α* and *HIF1B* (Fig. 6D). Thus, we hypothesis that the knockdown of SETDB1 may influence the translation of *HIF1Α*. To explore the underlying mechanisms by which SETDB1 affects HIF1α expression, we performed RNC-mRNA sequencing and found that loss of SETDB1 arrest translation ratio (TR) and ribosome nascent-chain complex (RNC) of *HIF1α* (Fig. 6E-H). These results suggest that SETDB1 regulates the expression of HIF1α at the translational level.

### SETDB1 regulates HIF1α translation via the mTOR-4EBP1 axis

**Fig. 7:**
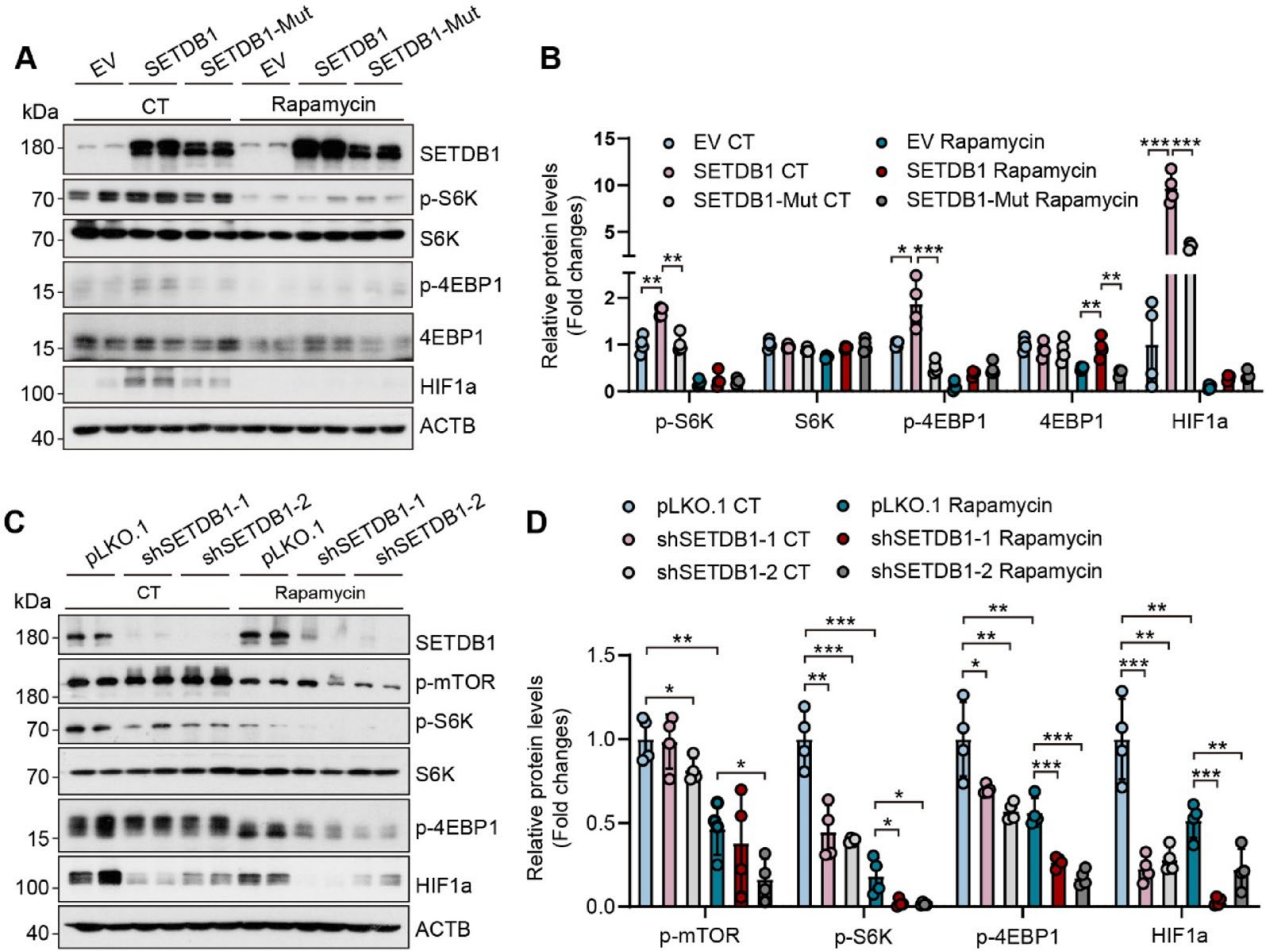
SETDB1regulates the translation of HIF1 through the mTOR-4EBP1 axis 2 A. **A-B.** Representative western blots (**A**) and quantitative results (**B**) of the indicated proteins in SETDB1 and SETDB1-Mut cells upon rapamycin treatment (data are presented as mean values ± SD, *p < 0.05, **p < 0.01, ***p < 0.001).**C-D.** Representative western blots (**C**) and quantitative results (**D**) of the indicated proteins in control and SETDB1 knockdown cells upon 1 µM rapamycin treatment for 6 h (data are presented as mean values ± SD, *p < 0.05, **p < 0.01, ***p < 0.001)

We next investigated the molecular mechanism for how SETDB1 regulates HIF1α expression. Several studies have reported that mTOR can be the upstream regulator affect the expression of HIF1α [34–36]. Western blot indicated that SETDB1 overexpression promoted phosphorylation of S6K and 4EBP1, and this effect was dependent on its methyltransferase activity (Figure S4C-D, Fig. 7A-B). Then we found that loss of SETDB1 inhibited phosphorylation of S6K and 4EBP1 (Figure S4E-F, Fig. 7C-D). These results suggest that SETDB1 regulates the translation of HIF1α through the mTOR-4EBP axis.

## Discussion

Gastric cancer (GC) remains one of the most common malignant tumors worldwide, and aberrant epigenetic modifications are increasingly recognized as key drivers of its initiation and progression. Among these epigenetic modifications, abnormal histone methylation is particularly associated with GC progression and research has mainly focused on H3 lysine 9 and 27 [37]. For example, H3K9 trimethylation has been positively correlated with advanced tumor stage, lymphovascular invasion, recurrence, and poor survival [38]. while H3K27 trimethylation has been linked with GC severity [39]. SETDB1, a histone methyltransferase responsible for di- and tri-methylation of H3K9, is highly expressed in multiple cancers, including GC [14]. Increasing evidence indicates that SETDB1 is highly expressed in majority of cancers including gastric cancer. Consistent with earlier reports, our data show that SETDB1 loss inhibits GC cell proliferation and migration, whereas overexpression exerts the opposite effect. Previous studies have demonstrated that the H1224K and C1226A mutants of SETDB1 showed impaired H3K9 methyltransferase activity [29]. Our experiments with the double mutant (H1224K/C1226A) revealed that migration was abolished without affecting the proliferation-promoting effect, underscoring functional divergence between these processes.

The unfolded protein response (UPR) is a cytoprotective program initiated by an excess of misfolded proteins in the ER lumen, associated with cancer progression [40, 41]. Although the relationship between SETDB1 and UPR has been explored in non-cancer contexts, it has rarely been investigated in oncology. Pasquarella et al. reported that in *Setdb1*-deficient pro-B cells, some ‘responsive’ murine leukemia virus (MLV) elements displayed strong transcriptional activity, which could trigger the unfolded protein response (UPR) [42]. Han and colleagues discovered inhibited Setdb1 translocation from the nucleus to the cytoplasm could led to decreases in Serpinh1, the accumulation of unfolded proteins and the elicitation of ER stress [43]. In our study, further studies should be done to elucidate whether SETDB1 directly regulates UPR-related genes.

c-MYC is an oncogene frequently implicated in gastric cancer and can promote the growth and proliferation of GC cells [44]. As a transcription factor, c-MYC also regulates cell cycle related genes including CCND1s and CKDs [45, 46]. The UPR and c-MYC are interconnected, with c-MYC influencing the UPR’s activation and the UPR, in turn, contributing to c-MYC ‘s oncogenic effects [47, 48]. In this study, we found that SETDB1 regulated c-MYC expression through the UPR pathway, suggest that SETDB1 may act as an upstream modulator connecting these two pathways. However, the inconsistency between cellular phenotype and molecular pathway, including possible post-translational regulation, feedback mechanisms, or other compensatory pathways that may contribute to the rescued cell survival induced by SETDB1 overexpression upon MitA treatment, even without obvious restoration of related protein levels.

Mammalian target of rapamycin (mTOR) plays a critical role in cancer development and progression by regulating cell growth, proliferation, survival, and metabolism [49, 50]. Previous studies have suggested an association between SETDB1 expression and the PI3K/AKT/mTOR pathway [51]. HIF1α, a transcription factor activated by hypoxia, plays a crucial role in tumor survival, angiogenesis and metastasis. In cancer, the mTOR and HIF1α pathways are intricately linked, with mTOR frequently acting as an upstream regulator of HIF1α expression and activity [36, 52, 53]. Our results show that SETDB1 activates mTOR and 4EBP1 to promote HIF1α translation, linking epigenetic regulation with hypoxia signaling in GC.

Accumulating evidence have shown the mechanism of interconnections and molecular links between mTOR signaling and UPR pathway in cancer progression. Constitutive mTORC1 activation contributes to ER-stress-induced apoptosis by loss of TSC1–TSC2 stimulates JNK [54, 55]. Furthermore, mTOR-mediated upregulation of protein synthesis induces the accumulation of misfolded or unfolded proteins in the ER lumen, which induces UPR. Reciprocally, ER stress regulates the PI3K/AKT/mTOR signaling pathway. Pharmacological induction of the UPR rapidly activates the PI3K/AKT/mTOR signaling axis [56–58]. Therefore, the cross-talk between the mTOR and UPR signaling pathways during cellular stress can affect cancer progression and may be involved in the pathogenesis and therapeutic outcome of cancer.

Collectively, our findings reveal a dual mechanistic role for SETDB1 in GC: SETDB1 modulated the cell cycle through UPR-mediated transcriptional activation of c-MYC, and activated the mTOR–4EBP1 axis to enhance HIF1α translation. These insights highlight SETDB1 as a potential therapeutic target not only in GC but also in other malignancies where similar pathways are active. Future studies should focus on the development of small-molecule inhibitors or RNA-based strategies capable of selectively disrupting SETDB1’s context-specific functions.

## Conclusion

In summary, we identified SETDB1 as an epigenetic regulator that is markedly upregulated in gastric cancer (GC) and serves as a potential therapeutic target. In this study, SETDB1 promoted both proliferation and migration of GC cells, with migration dependent on its methyltransferase activity but proliferation largely independent of it. Mechanistically, SETDB1 modulated the cell cycle through UPR-mediated transcriptional activation of c-MYC, and activated the mTOR–4EBP1 axis to enhance HIF1α translation.

## Supplementary Information

Supplementary Material 1

Supplementary Material 2

## Data Availability

All data supporting this study are available from the corresponding authors upon reasonable request.
