# Supplementary material for: SETDB1 promotes gastric cancer progression via UPR and mTOR pathway
Source: Cancer Cell Int. 2026 Apr 11;26:204. doi: 10.1186/s12935-026-04296-1 (PMC13214091; doi:10.1186/s12935-026-04296-1)
Supplement: Supplementary file 1 — Supplementary Material 1 [file 12935_2026_4296_MOESM1_ESM.pptx]

## Slide 1
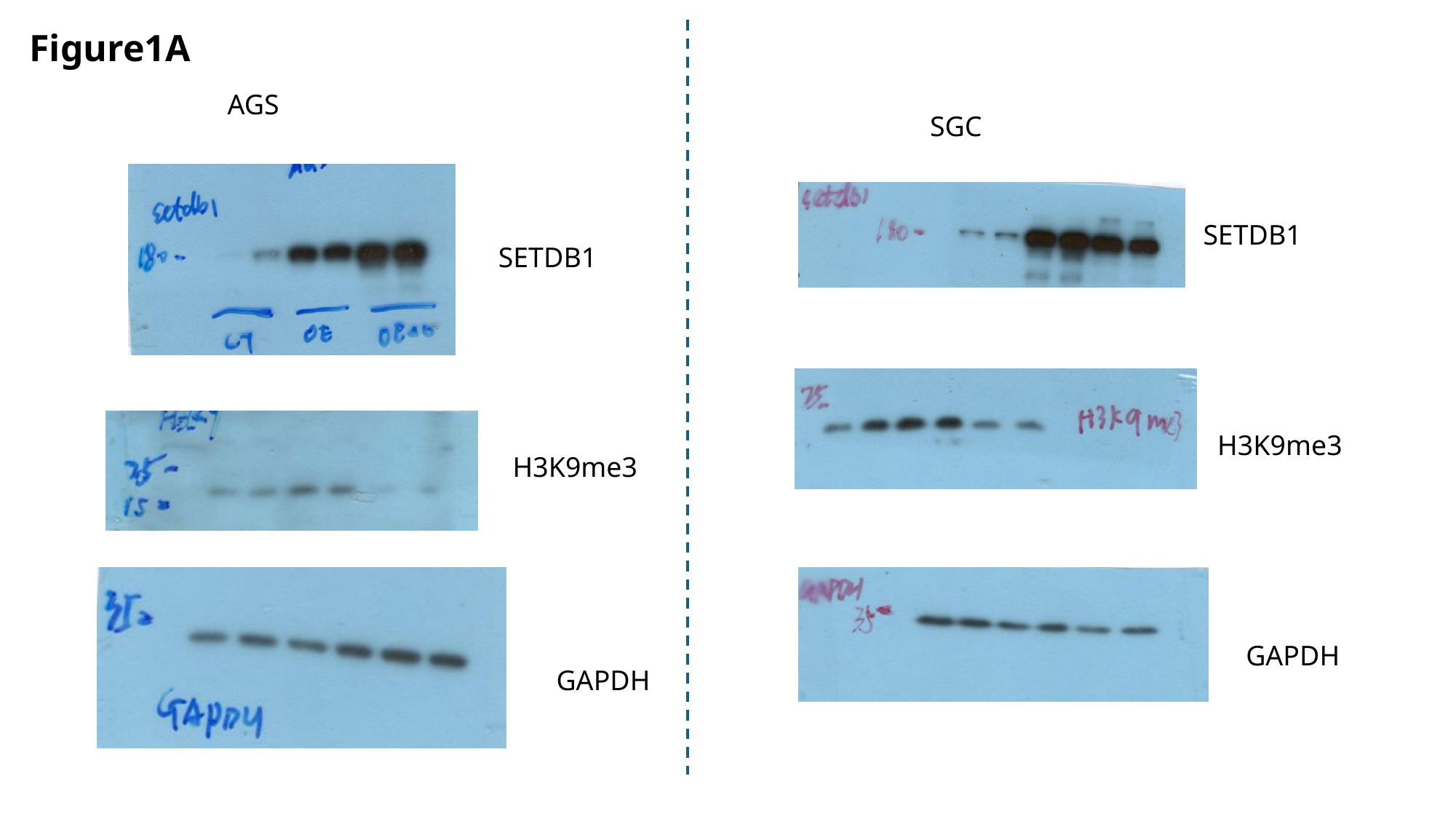

Figure1A
AGS
SGC
SETDB1
SETDB1
H3K9me3
H3K9me3
GAPDH
GAPDH

## Slide 2
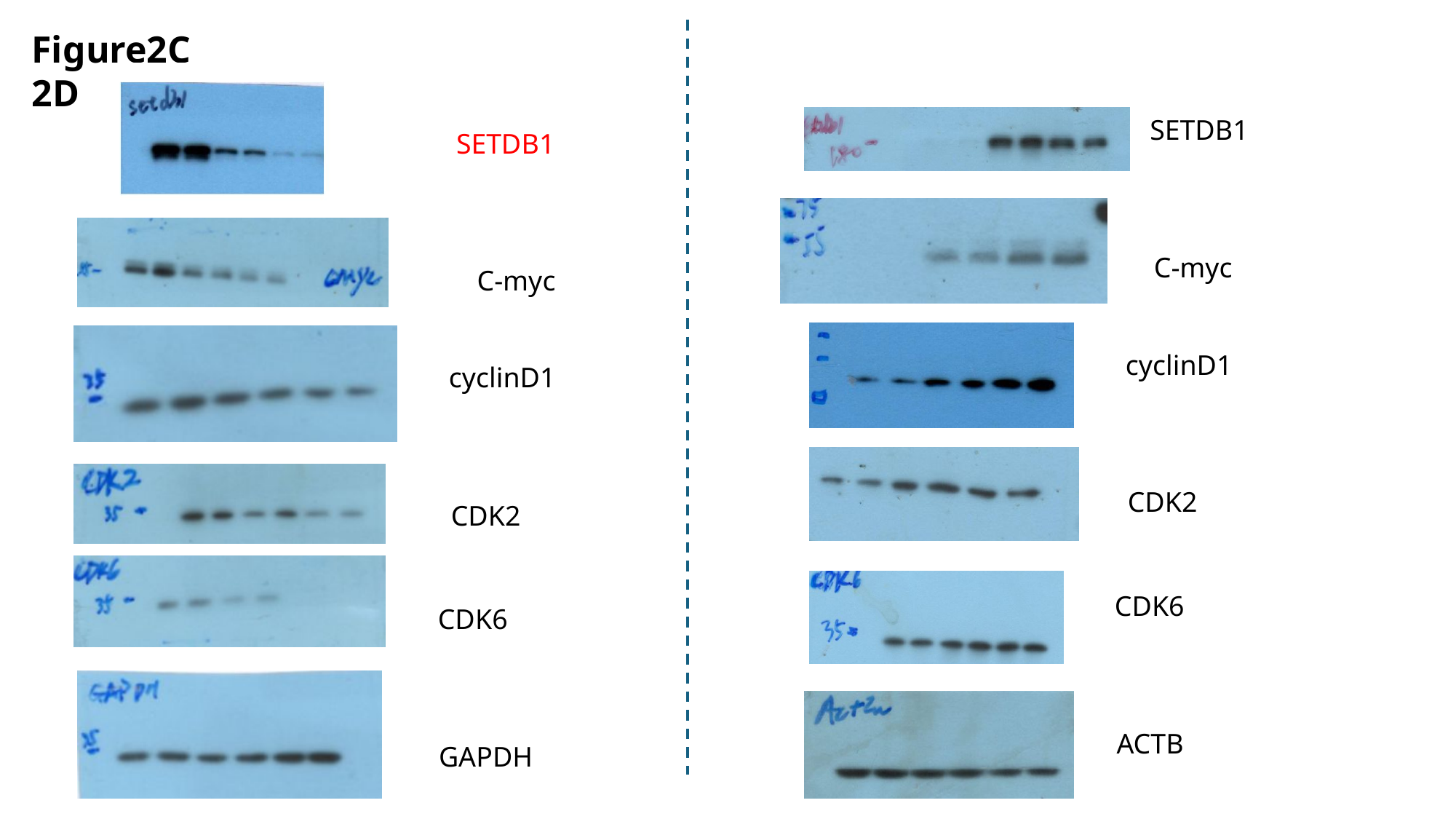

Figure2C 2D
SETDB1
SETDB1
C-myc
C-myc
cyclinD1
cyclinD1
CDK2
CDK2
CDK6
CDK6
ACTB
GAPDH

## Slide 3
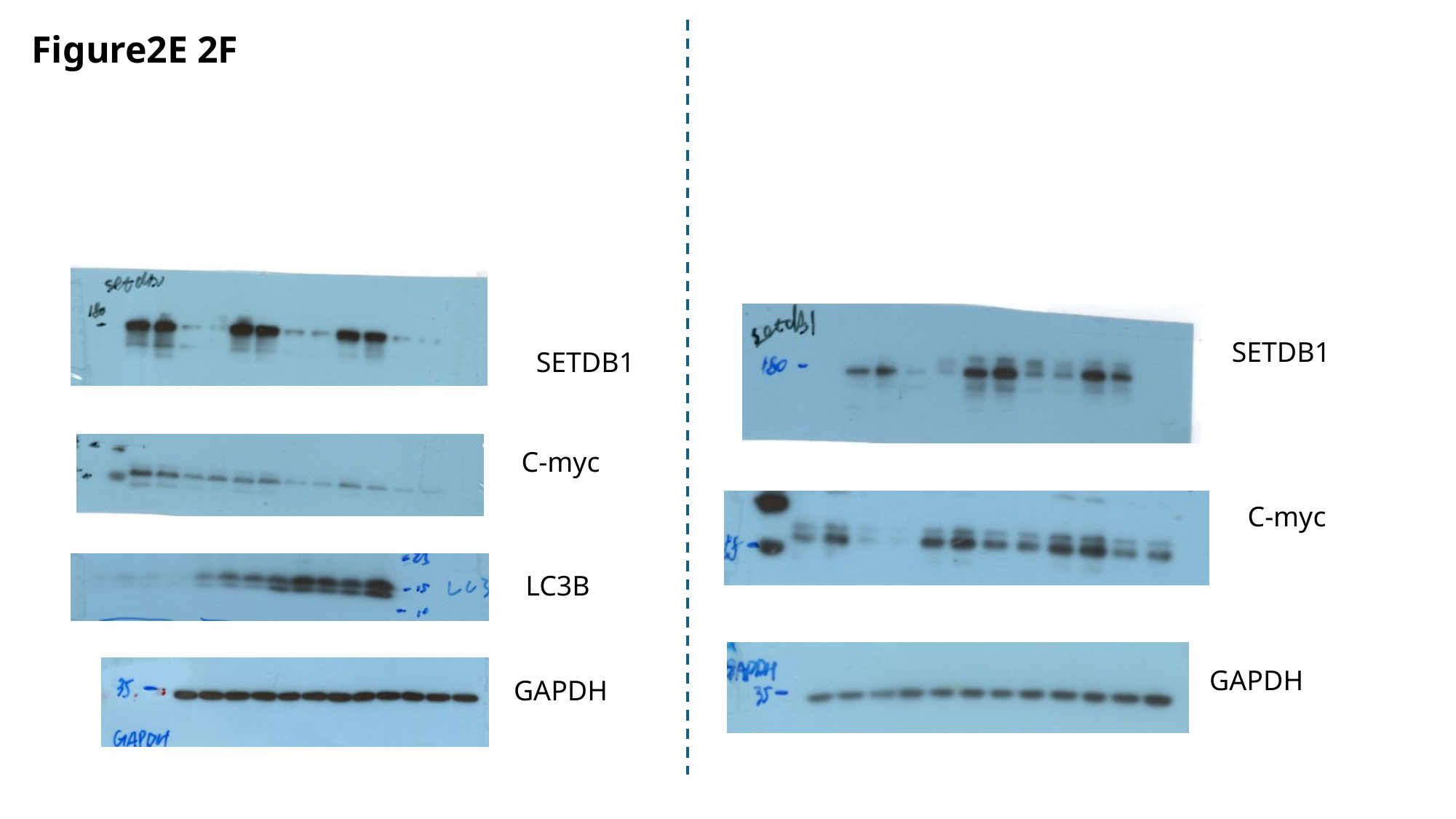

Figure2E 2F
SETDB1
SETDB1
C-myc
C-myc
LC3B
GAPDH
GAPDH

## Slide 4
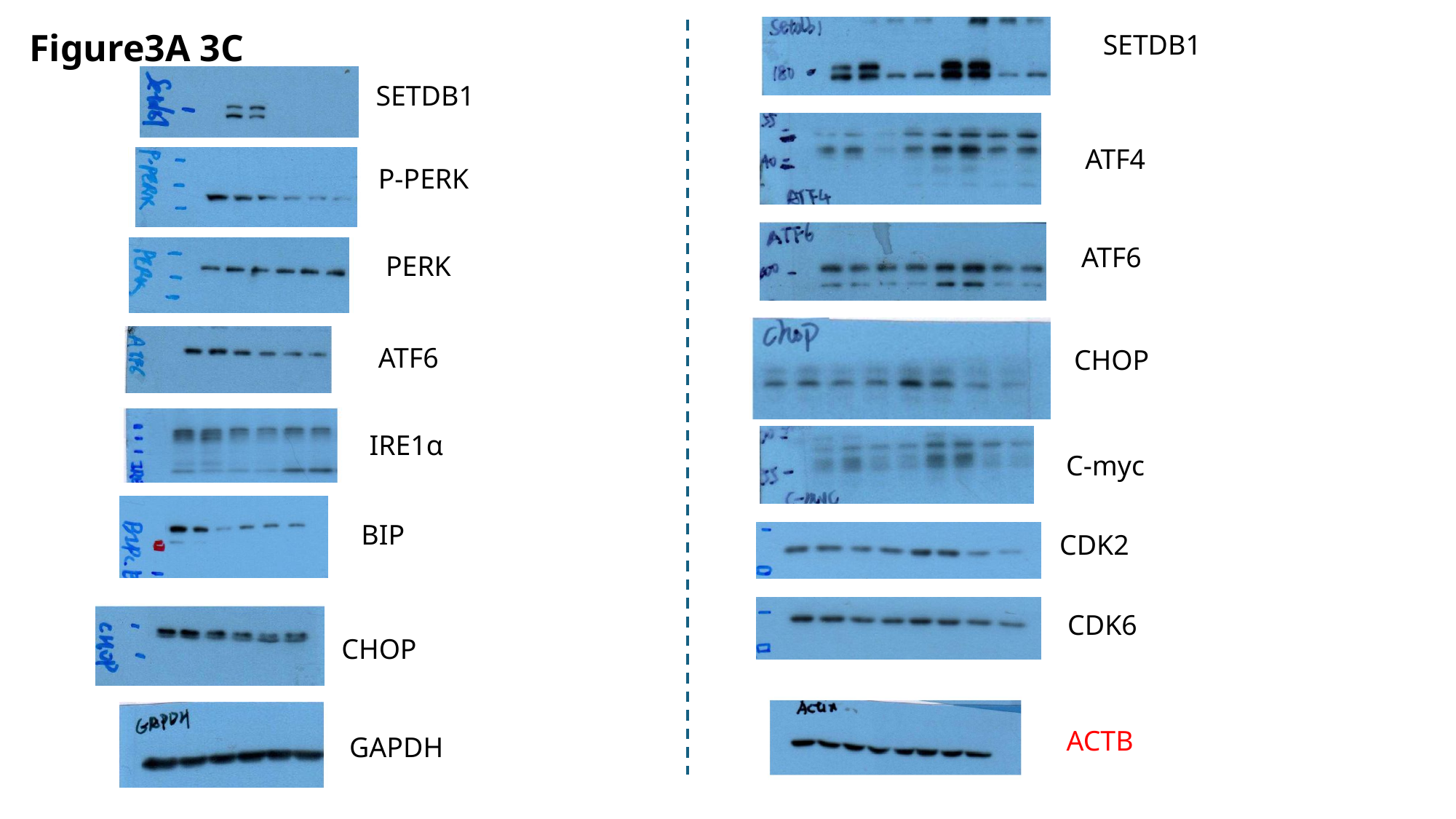

Figure3A 3C
SETDB1
SETDB1
ATF4
P-PERK
ATF6
PERK
ATF6
CHOP
IRE1α
C-myc
BIP
CDK2
CDK6
CHOP
ACTB
GAPDH

## Slide 5
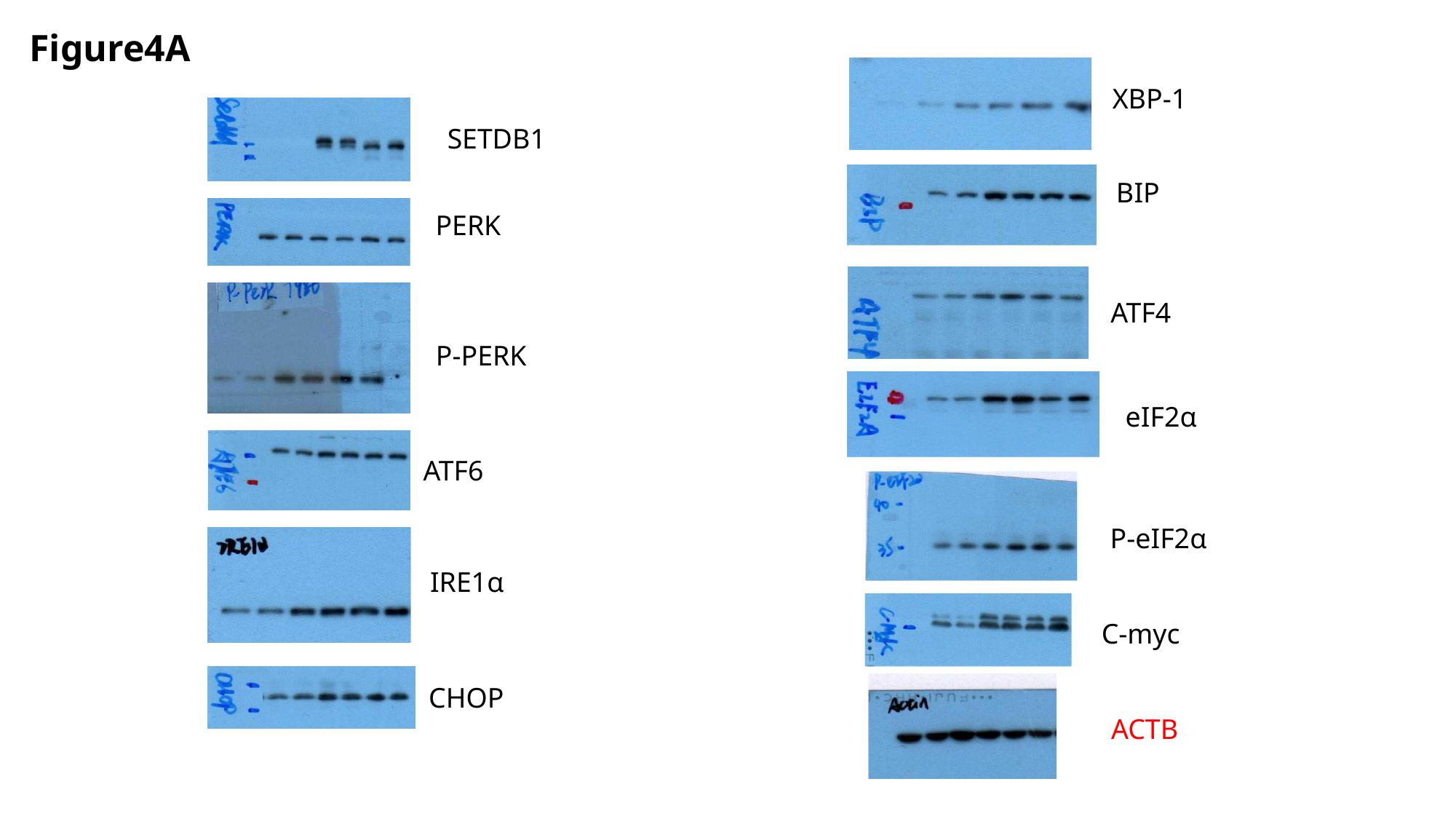

Figure4A
XBP-1
SETDB1
BIP
PERK
ATF4
P-PERK
eIF2α
ATF6
P-eIF2α
IRE1α
C-myc
CHOP
ACTB

## Slide 6
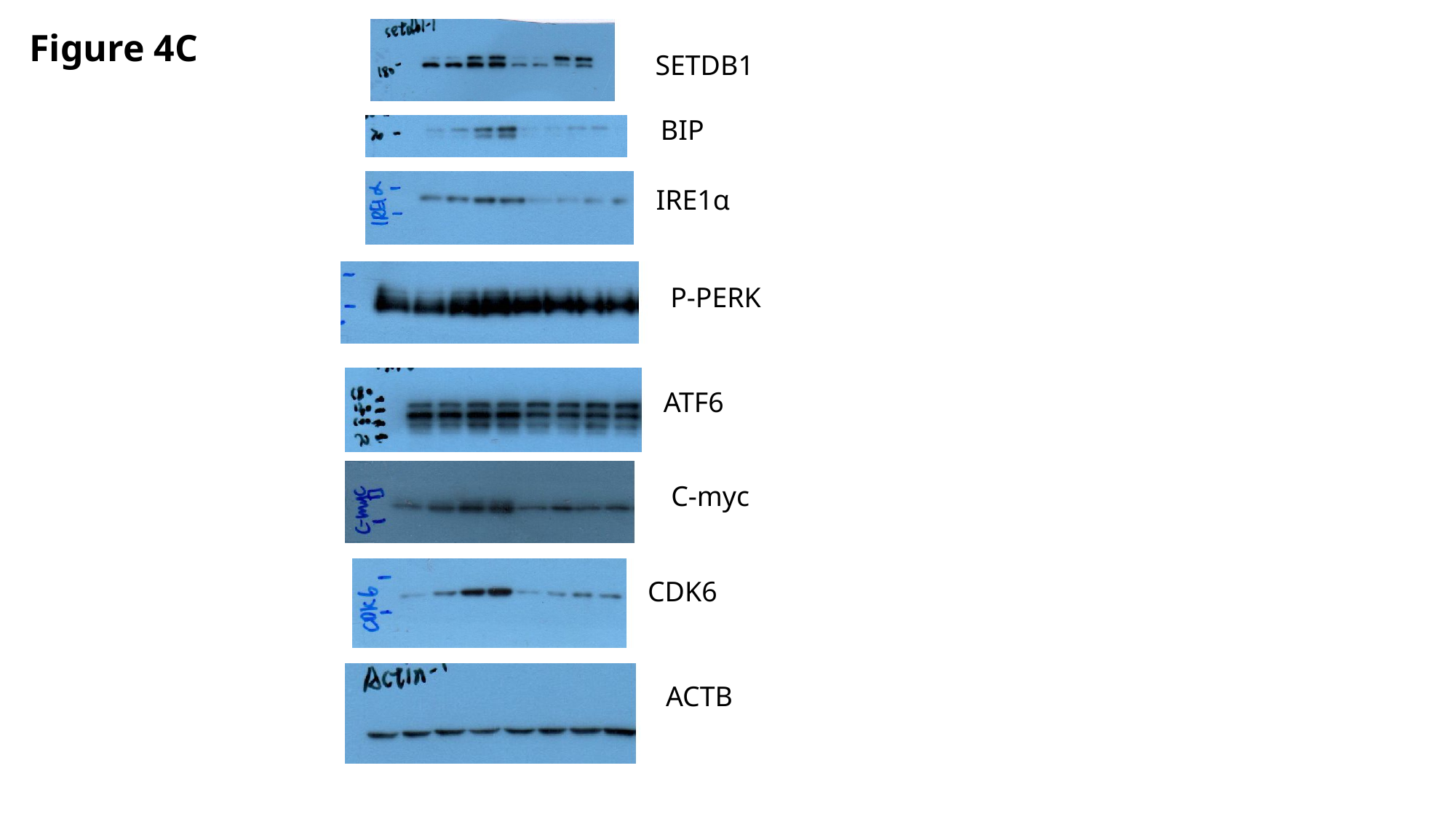

Figure 4C
SETDB1
BIP
IRE1α
P-PERK
ATF6
C-myc
CDK6
ACTB

## Slide 7
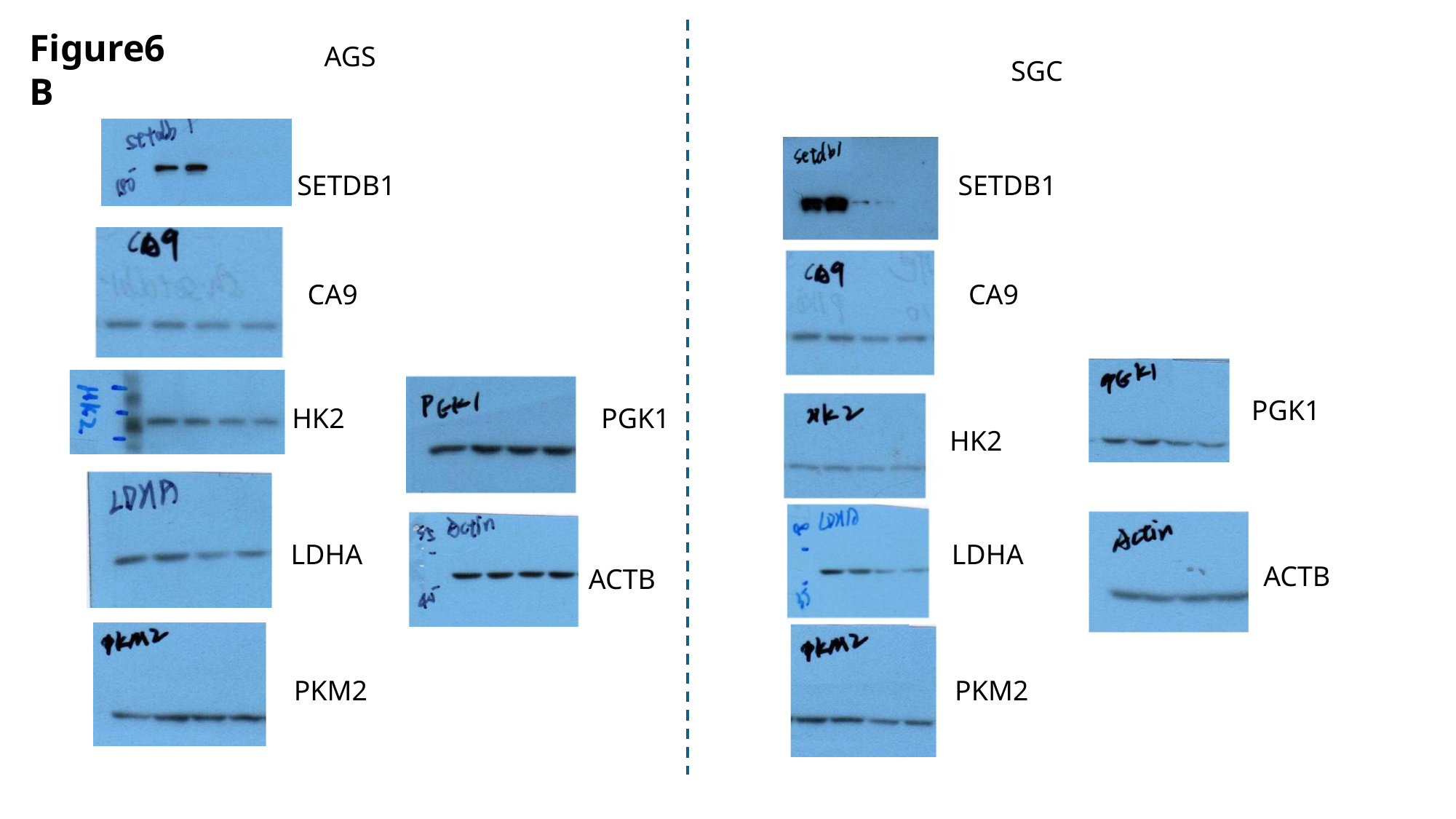

Figure6B
AGS
SGC
SETDB1
SETDB1
CA9
CA9
PGK1
HK2
PGK1
HK2
LDHA
LDHA
ACTB
ACTB
PKM2
PKM2

## Slide 8
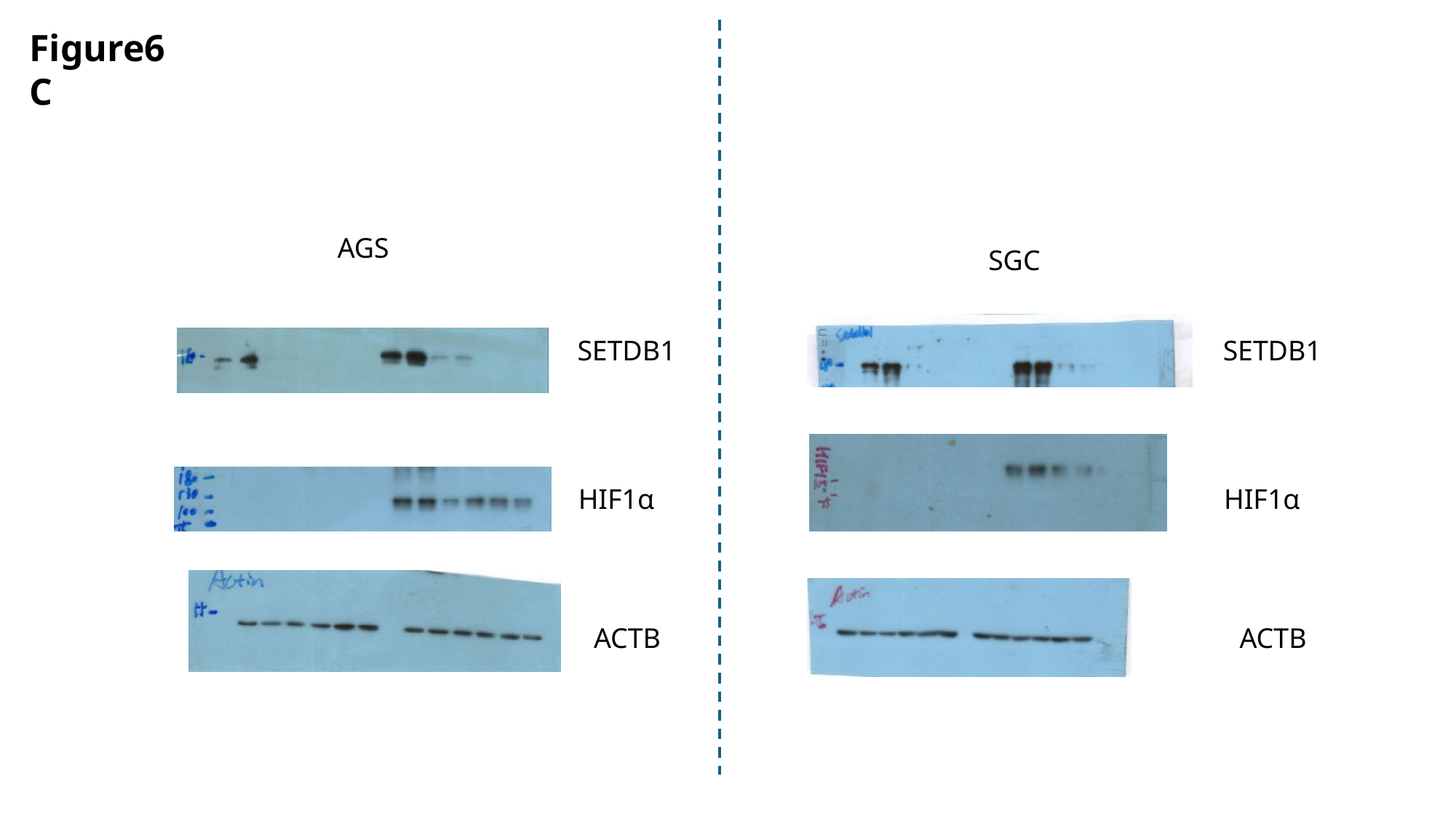

Figure6C
AGS
SGC
SETDB1
SETDB1
HIF1α
HIF1α
ACTB
ACTB
