# Supplementary material for: SETDB1 promotes gastric cancer progression via UPR and mTOR pathway
Source: Cancer Cell Int. 2026 Apr 11;26:204. doi: 10.1186/s12935-026-04296-1 (PMC13214091; doi:10.1186/s12935-026-04296-1)
Supplement: Supplementary file 2 — Supplementary Material 2 [file 12935_2026_4296_MOESM2_ESM.docx]

**SETDB1 promotes gastric cancer progression via UPR and mTOR pathway**

Jing Qiao ^1^, Shijie Lin ^2^, Yeju Li ^2^, Xiaoyang Yue ^2^, Yanyan Liu ^2^, Huajian Tian ^2^, Jianshuang Li ^2,4^, Junyang Tan ^2,3*^

^1^ The Affiliated Qingyuan Hospital (Qingyuan People’s Hospital), Guangzhou Medical University, Qingyuan, Guangdong, 511518, China.

^2^ State Key Laboratory of Bioactive Molecules and Druggability Assessment, Guangdong Basic Research Center of Excellence for Natural Bioactive Molecules and Discovery of Innovative Drugs, College of Life Science and Technology, Jinan University, Guangzhou, Guangdong, 510632, China.

^3^ Department of Anesthesiology and Clinical Research Institute, The First Affiliated Hospital of Jinan University, Guangzhou, Guangdong, 510630, China.

^4^ Department of Orthopaedics, Guangzhou Red Cross Hospital, Faculty of Medical Science, Jinan University Guangzhou 510220, Guangdong, China.

* Corresponding authors

Junyang Tan: [junyangtan@jnu.edu.cn](mailto:wangwenjun@jnu.edu.cn)

**Keywords:** gastric cancer, SETDB1, histone H3K9 methyltransferase, unfolded protein response, mTOR

**Supplementary figures and legends**


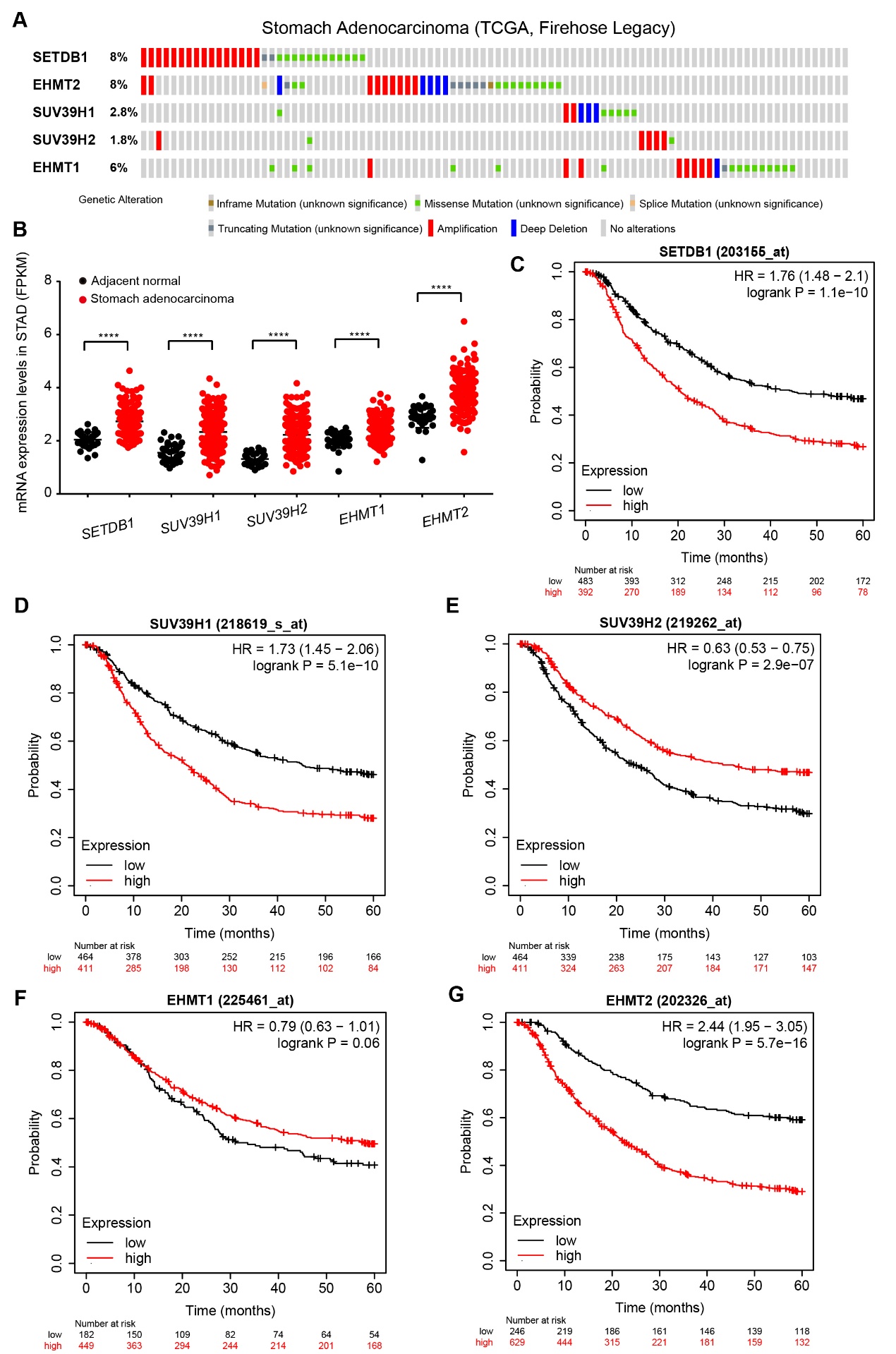


**Figure S1 Expression and prognosis of five methyltransferases in gastric cancer**

**A**. The proportion and distribution of samples with alterations of 5 H3K9 methyltransferase genes (SETDB1, EHMT2, SUV39H1, SUV39H2, EHMT1). **B**. Analysis of 5 H3K9 methyltransferase genes expression in adjacent normal and stomach adenocarcinoma from TCGA database (data are presented as mean values ± SD, ****p* < 0.001). **C-G.** Survival analysis from patients with low or high expression levels of SETDB1, EHMT2, SUV39H1, SUV39H2 and EHMT1 in GC from TCGA database.


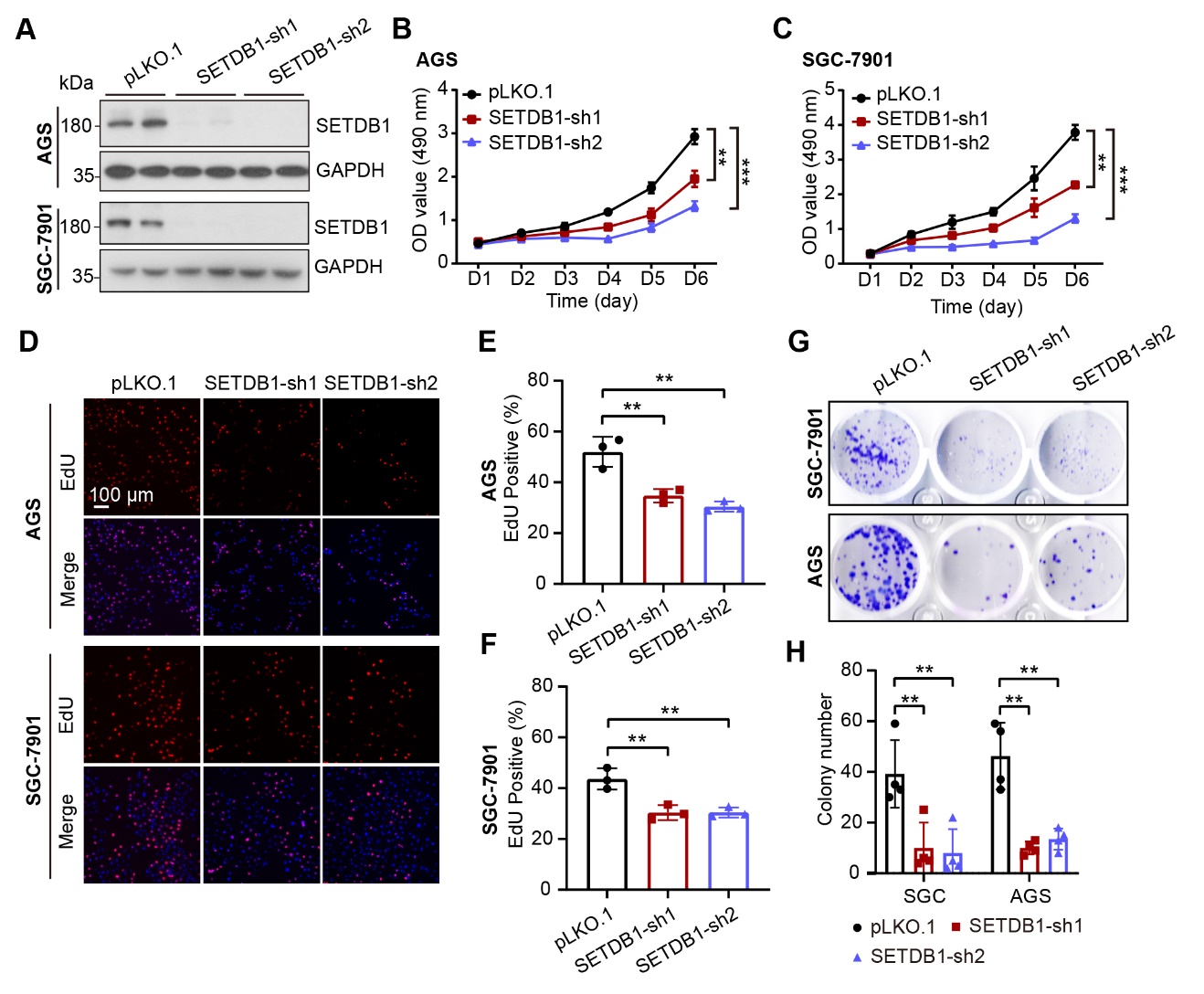


**Figure S2 Loss of SETDB1 inhibits the proliferation of GC cells**

**A.** Representative western blot results of the knockdown efficiency of SETDB1 in AGS and SGC-7901 cell lines. **B-C.** CCK8 assay shows the cell survival of control and SETDB1 knockdown in AGS and SGC-7901 cell lines (data are presented as mean values ± SD, ***p* < 0.01, ****p* < 0.001).  **D-F.** Representative images of EdU staining (**D**) and quantification results (**E-F**) in AGS and SGC-7901 cells. The images display EdU staining (red color) merged with DAPI staining (blue color) (Scale bar = 100 μm; data are presented as mean values ± SD, ***p* < 0.01). **G-H.** Colony formation analysis of control and SETDB1 knockdown in AGS and SGC-7901 cell lines (data are presented as mean values ± SD, ***p* < 0.01).


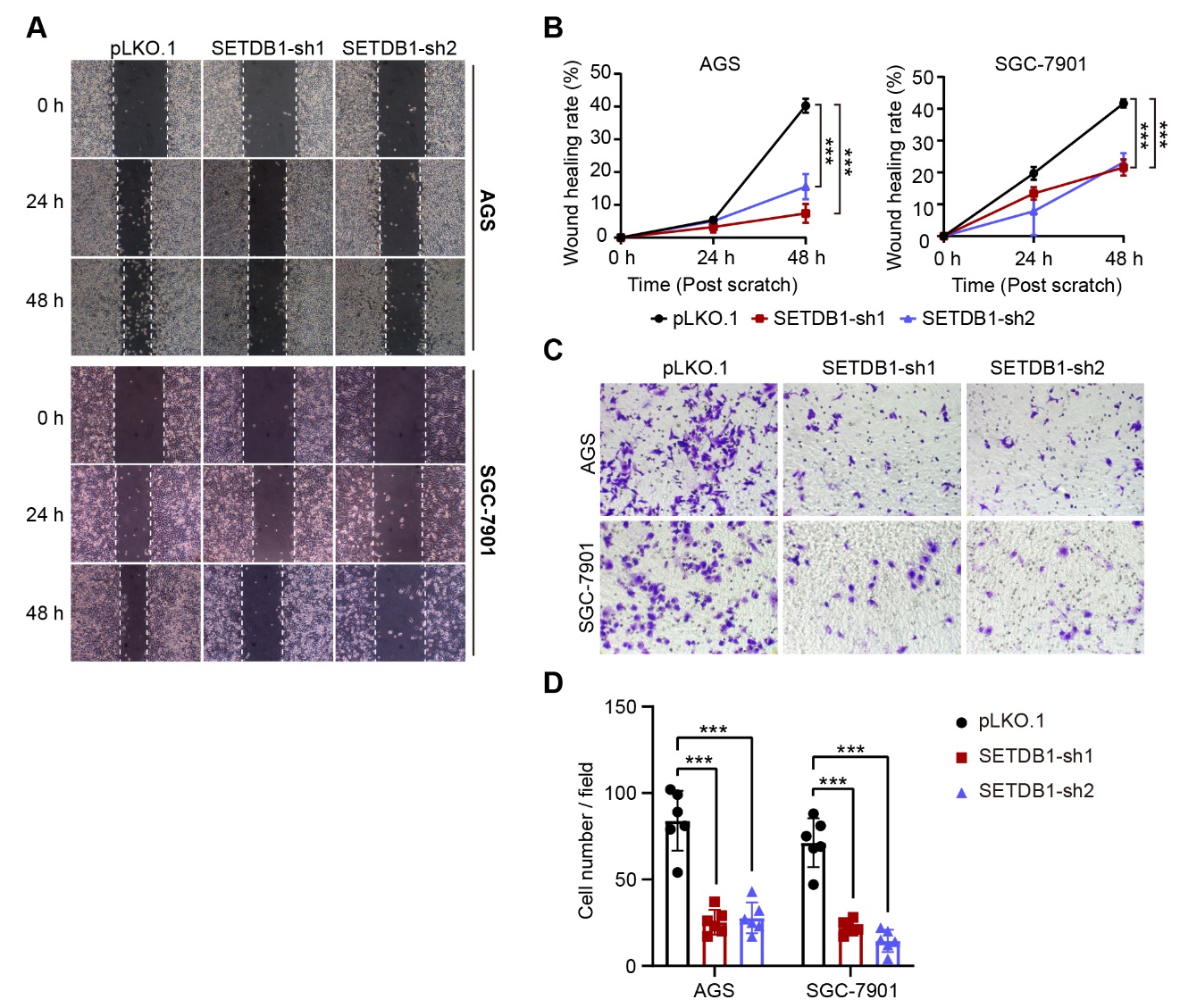


**Figure S3 Loss of SETDB1 inhibits the migration of GC cells**

**A-B.** Representative images (**A**) and wound healing rate (**B**) of the wound healing assay in control and SETDB1 knockdown AGS and SGC-7901 cells (Scale bar = 200 μm; data are presented as mean values ± SD, ****p* < 0.001). **C-D.** Transwell assay (**C**) and statistical results (**D**) in control and SETDB1 knockdown AGS and SGC-7901 cells (data are presented as mean values ± SD, ****p* < 0.001).


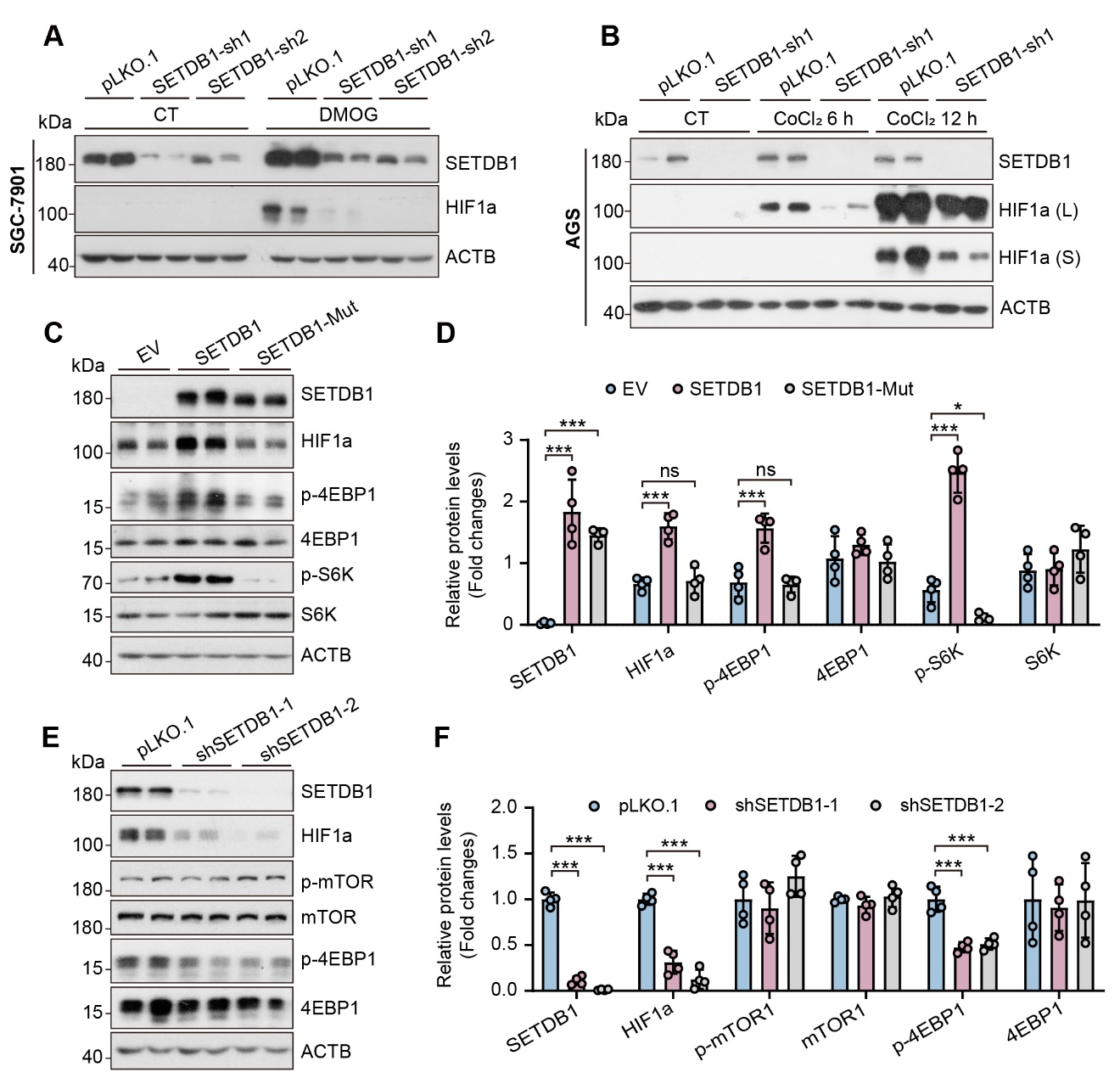


**Figure S4 Loss of SETDB1 inhibits the migration of GC cells**

**A.** Representative western blots of the indicated proteins of control and SETDB1 knockdown upon 100 μM DMOG treatment for 6 hours. **B.** Representative western blots of the indicated proteins of control and SETDB1 knockdown upon 50 μM CoCl_2_ treatment for 12 hours. **C-D.** Representative western blots (**C**) and quantitative results (**D**) of the indicated proteins in SETDB1 and SETDB1-Mut cells (data are presented as mean values ± SD, **p* < 0.05, ****p* < 0.001). **E-F.** Representative western blots (**E**) and quantitative results (**F**) of the indicated proteins in control and SETDB1 knockdown cells (data are presented as mean values ± SD, ****p* < 0.001).
